# Gender bias, social bias, and representation: 70 years of B^*H*^ollywood

**DOI:** 10.1016/j.patter.2022.100442

**Published:** 2022-02-11

**Authors:** Kunal Khadilkar, Ashiqur R. KhudaBukhsh, Tom M. Mitchell

## Main text

(Patterns *3*, 100409, February 11, 2022)

In the originally published version of this article, Table 9 and Figure 11 had incorrect values due to an inadvertent error by the authors. In Table 9, for city mentions in old movies (1950-1969), Bombay/Mumbai should read 51, not 55; Delhi should read 27, not 30; Agra should read 6, not 50; Mathura should read 5, not 6; Kolkata/Calcutta (23), Madras/Chennai (10), and Simla/Shimla (6) should be included; and Daman, Jalgaon, and Allahabad should be removed. For mid movies (1970-1999), Bombay/Mumbai should read 68, not 73; Delhi should read 45, not 46; Pune should read 8, not 9; Bangalore/Bengaluru should read 7, not 8; Nagpur should read 6, not 7; Kolkata/Calcutta (18), Simla/Shimla (12), Madras/Chennai (10) should be included; and Agra, Daman, and Rampur should be removed. For new movies (2000-2020), Bombay/Mumbai should read 83, not 91; Delhi should read 52, not 63; Bangalore/Bengaluru should read 9, not 11; Pune should read 8, not 10; Lucknow should read 7, not 8; Hyderabad should read 6, not 9; Kolkata/Calcutta (9) and Madras/ Chennai (6) should be included; and Agra and Chandigarh should be removed. The title of Table 9 has also been updated to include the full year (e.g., 1950-1969) rather than a shortened form (1950-69).

In Figure 11C, “No representation” should read “Least/No representation,” and “States never mentioned in 70 years” should read “Least/No State Mentions in the last 70 years”. Additionally, the legend (C) should now read “States with least or no representation (less than 0.2% movies in the entire corpus) in our corpus in the last 70 years” rather than “States never mentioned in our corpus in the last 70 years.”

In the text, the sentence “As of 2021, India has 29 states and seven union territories” was incorrect and should read “As of 2021, India has 28 states and eight union territories.” Also, regarding under-representation in northeastern states, we inadvertently included Manipur and Tripura in the sentence “In fact, there have been zero mentions of the states of Manipur, Arunachal Pradesh, Meghalaya, Tripura, and Mizoram in over 700 movies across 70 years.” It should instead read “In fact, there have been zero mentions of the states of Arunachal Pradesh, Meghalaya, and Mizoram in over 700 movies across 70 years.”

These errors have now been corrected online, the overall broad conclusions of the paper remain unaffected by the updated results, and the authors sincerely apologize for these errors.

**Figure:**
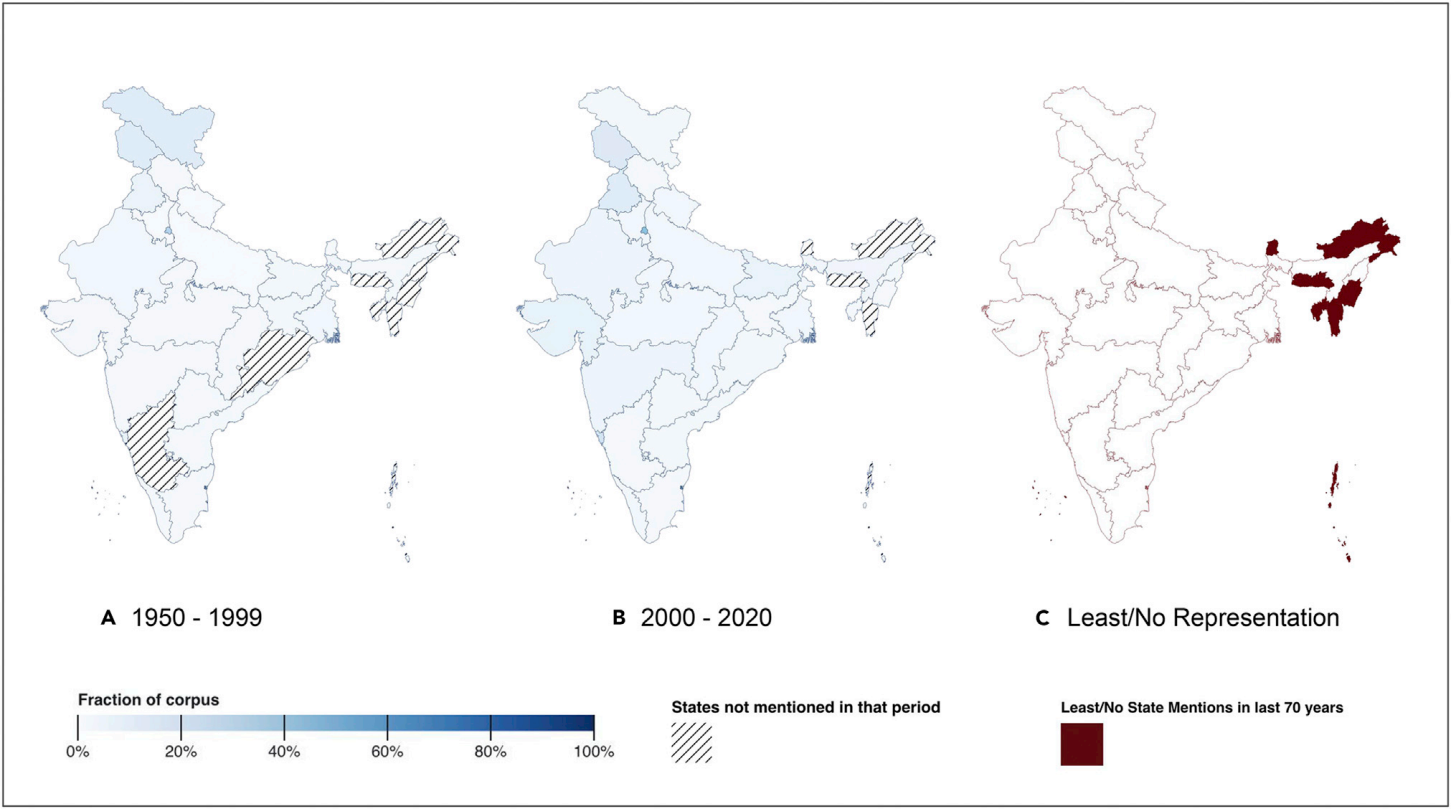
Figure 11. Geographic representation in Bollywood movies (corrected)

**Figure:**
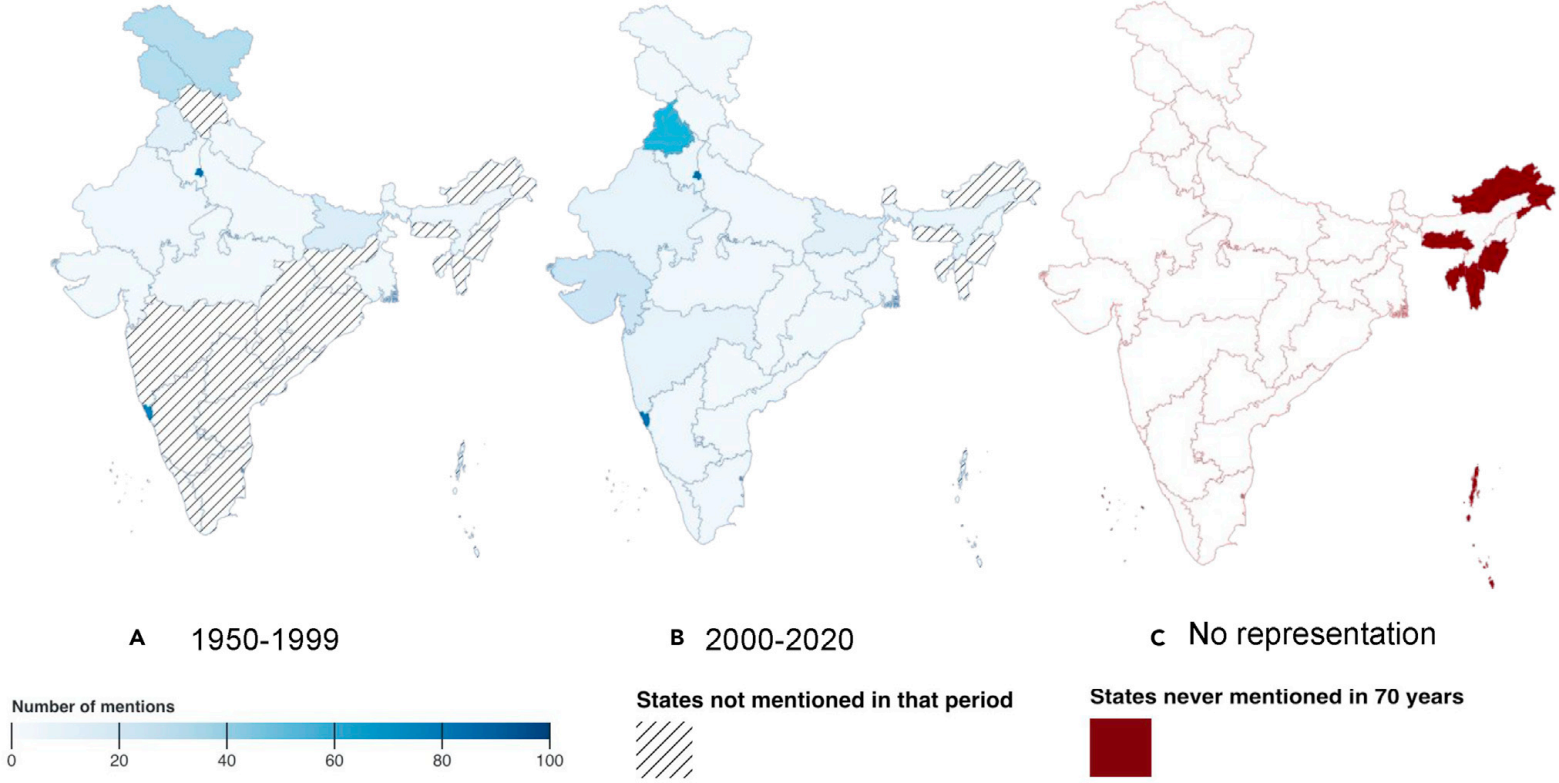
Figure 11. Geographic representation in Bollywood movies (original)

