# Gender bias, social bias, and representation in Bollywood and Hollywood

**DOI:** 10.1016/j.patter.2022.100486

**Published:** 2022-04-08

**Authors:** Kunal Khadilkar, Ashiqur R. KhudaBukhsh, Tom M. Mitchell

## Main text

(Patterns *3*, 100409; February 11, 2022)

In this article’s original title, the authors used the term “BHollywood” to refer to both Bollywood and Hollywood. In order to ensure the title is as clear as possible and avoid misunderstandings, the title now refers to Bollywood and Hollywood as separate entities. We have now updated the title in the article online as well as in the title of a Correction to the article that we published previously.

Also, due to an error in Production, in several tables’ column headings, the subscripts “$D_{bolly}^{new}$” and “$D_{holly}^{new}$” were inadvertently written as “$D_{bolly}^{old}$” and “$D_{holly}^{old}$,” respectively. In Table 2, the third column from the right previously read “$D_{bolly}^{old}$” and now reads “$D_{bolly}^{new}$,” and the rightmost column previously read “$D_{holly}^{old}$” and now reads “$D_{holly}^{new}$”; in Table 11, the rightmost column has now been corrected from “$D_{bolly}^{old}$” to “$D_{bolly}^{new}$”; and in Table 12, the center column has now been corrected from “$D_{bolly}^{old}$” to “$D_{holly}^{old}$,” and the right column from “$D_{bolly}^{old}$” to “$D_{holly}^{new}$.” The journal team regrets these errors and apologizes for any confusion they may have caused.

