# Supplementary material for: Gender bias, social bias, and representation: 70 years of BHollywood
Source: Patterns (N Y). 2021 Dec 9;3(2):100409. doi: 10.1016/j.patter.2021.100409 (PMC8848024; doi:10.1016/j.patter.2021.100409)
Supplement: Document S1. AAAI 2021 student abstract [file mmc1.pdf]

Patterns, Volume *3*

Supplemental information

Gender bias, social bias, and representation: 70 years of B^{H}ollywood

Kunal Khadilkar, Ashiqur R. KhudaBukhsh, and Tom M. Mitchell

PRELIMINARY PREPRINT VERSION: DO NOT CITE
The AAAI Digital Library will contain the published
version some time after the conference.

An Unfair Affinity Toward Fairness: Characterizing 70 Years of Social Biases in B^{H}ollywood (Student Abstract)

Kunal Khadilkar[1] and Ashiqur R. KhudaBukhsh [1]
[1] Carnegie Mellon University
kkhadilk@cs.cmu.edu, akhudabu@cs.cmu.edu

Abstract

Bollywood, aka the Mumbai film industry, is one of the biggest movie industries in the world with a current movie market share of worth 2.1 billion dollars and a target audience base of 1.2 billion people. While the entertainment impact in terms of lives that Bollywood can potentially touch is mammoth, no NLP study on social biases in Bollywood content exists. We thus seek to understand social biases in a developing country through the lens of popular movies. Our argument is simple – popular movie content reflects social norms and beliefs in some form or shape. We present our preliminary findings on a longitudinal corpus of English subtitles of popular Bollywood movies focusing on (1) social bias toward a fair skin color (2) gender biases, and (3) gender representation. We contrast our findings with a similar corpus of Hollywood movies. Surprisingly, we observe that much of the biases we report in our preliminary experiments on the Bollywood corpus, also gets reflected in the Hollywood corpus.

Introduction

On a diachronic corpus of popular entertainment, what types of social biases can we analyze and detect? In this paper, we analyze a curated corpus of film subtitles from the Bollywood and Hollywood film industry, spread over 70 years and present our preliminary findings on three aspects: (1) social bias toward a fair skin color; (2) gender biases; and (3) gender representation. While studies analyzing gender stereotypes across different languages (Lewis and Lupyan 2020) and detecting bias in word embeddings (Garg et al. 2018) exist, Bollywood in NLP literature has received little focus (Khan and Taylor 2018; Madaan et al. 2018) and no existing work has considered the rich data set of subtitles contrasting the findings with a Hollywood subtitles corpus.

In Figure 1, what do the prominent presence of negative adjectives such as `wanton` in old Bollywood movies (see, Figure 1(a)) juxtaposed with the presence of positive verbs such as `respect` (see, Figure 1(b)) tell us? In this paper, we explore a recent technique (Palakodety, KhudaBukhsh, and Carbonell 2020) used to mine political insights in a novel context of uncovering social biases. Through a series of cloze tests (Taylor 1953) on a language model (Devlin et al. 2019) fine-tuned on our data sets, we present our preliminary findings.

Copyright © 2021, Association for the Advancement of Artificial Intelligence (www.aaai.org). All rights reserved.

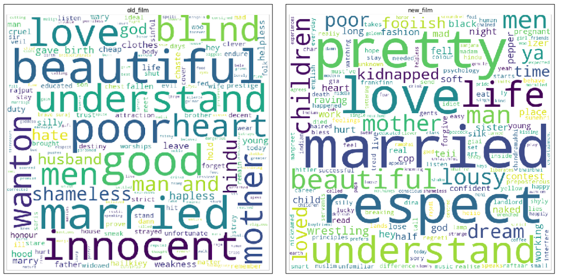

(a) Old Bollywood (b) Recent Bollywood

Figure 1: Word cloud visualizations for the five word contexts containing utterances of 'woman/women'.

Our data sets: We construct the following two data sets.
1. Bollywood movies, $\mathcal{D}_{bolly}$: We consider movies spanning seven decades (1950–2020). For each decade, we retrieved subtitles (Lison and Tiedemann 2016) of 40 top-grossing movies (280 total films), based on earnings documented by IMDb (Internet Movie Database).
2. Hollywood movies, $\mathcal{D}_{holly}$: Similarly, we considered 40 top-grossing movies from each of the seven decades (280 total films). Overall, $\mathcal{D}_{bolly}$ and $\mathcal{D}_{holly}$ consist of 542,768 and 488,498 dialogues, respectively.

For a subset of our analyses, we divide our corpus into two buckets: (1) Films from 1950 to 1970 ($\mathcal{D}^{old}_{bolly}$ and $\mathcal{D}^{old}_{holly}$); and (2) Films from 2000 to 2020 ($\mathcal{D}^{new}_{bolly}$ and $\mathcal{D}^{new}_{holly}$).

Results

Pronouns as a Proxy for Representation: Following extensive literature on gendered pronouns' relative distributions and their implications (Twenge, Campbell, and Gentile 2012), we consider a simple measure of gender representation: relative occurrence of pronouns of each gender (Men: he, him. Women: she, her). Let $\mathcal{N}_w$ denote the number of times a token w appears in a corpus. We define *Male Pronoun Ratio (MPR)* as follows:
$MPR = \frac{\mathcal{N}_{he}+\mathcal{N}_{him}}{\mathcal{N}_{he}+\mathcal{N}_{him}+\mathcal{N}_{she}+\mathcal{N}_{her}} * 100$. Figure 2 plots *MPR* of our decade-wise movie data sets and contrasts with *MPR* computed using Google Ngrams. Our results indicate that even now, both Bollywood and Hollywood exhibit compa-

Probe	$\texttt{BERT}_{\text{base}}$	$\texttt{BERT}_{\mathcal{D}^{old}_{bolly}}$	$\texttt{BERT}_{\mathcal{D}^{new}_{bolly}}$	$\texttt{BERT}_{\mathcal{D}^{old}_{holly}}$	$\texttt{BERT}_{\mathcal{D}^{new}_{holly}}$
$cloze_1$	soft, beautiful, pale, tanned, smooth	fair, no, pale, tanned, tan	fair, tanned, golden, smooth, pale	fair, pale, blue, golden, gold	fair, pale, tanned, golden, dark
$cloze_2$	woman, man, widow, *doctor*, slave, *soldier*, bachelor, *merchant*, *farmer*, *lawyer*, servant	prostitute, servant, woman, slave, bachelor, *doctor*, *lawyer*, man, widow, maid, *worker*	*doctor*, woman, servant, *lawyer*, maid, man, *nurse*, *teacher*, *gardener*, lady, hindu	woman, slave, servant, *nurse*, lady, man, *teacher*, *lawyer*, peasant, maid, wife	woman, *lawyer*, *doctor*, *nurse*, *teacher*, man, *writer*, *secretary*, prostitute, professional, *carpenter*
$cloze_3$	man, *soldier*, gentleman, *farmer*, *merchant*, woman, slave, bachelor, *doctor*, *carpenter*, servant	man, *gentleman*, bachelor, *lawyer*, servant, *doctor*, *farmer*, *worker*, *craftsman*, slave, criminal	*doctor*, *lawyer*, *policeman*, man, *farmer*, bachelor, *gardener*, servant, *soldier*, *mechanic*, *builder*	*carpenter*, *policeman*, *lawyer*, *soldier*, *farmer*, *gentleman*, servant, man, peasant, slave, *doctor*	man, *lawyer*, *soldier*, *doctor*, *carpenter*, *gentleman*, *farmer*, *clergyman*, *writer*, *craftsman*, *minister*

Table 1: Cloze test results. Predicted tokens are ranked by decreasing probability. Positive and negative words are italicized and underlined, respectively. Our completion results using BERT indicate presence of social and gender biases.

rable skew in gendered pronoun usage.

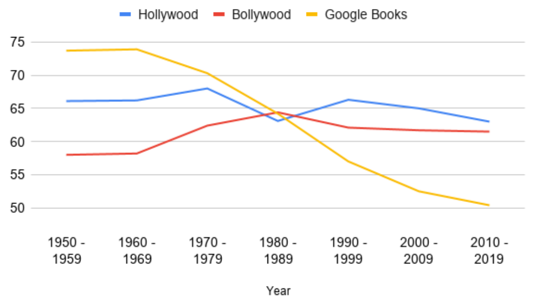

Figure 2: MPR in $\mathcal{D}_{bolly}$ and $\mathcal{D}_{holly}$

Cloze Test Using Language Models: When presented with a sentence (or a sentence stem) with a missing word, a cloze task (Taylor 1953) is essentially a fill-in-the-blank task. For instance, in the following cloze task: *In the* [MASK], *it is very sunny*, summer is a likely completion for the missing word. Given a cloze test, BERT, a well-known language model (Devlin et al. 2019), outputs a series of token ranked by probability. In fact, in the above cloze test, the top three tokens (ranked by probability) predicted by $\texttt{BERT}_{base}$ are: summer, winter and spring. Recent lines of research has explored BERT's masked query prediction for (1) knowledge base extraction (Petroni et al. 2019) and (2) mining political insights (Palakodety, KhudaBukhsh, and Carbonell 2020).

Following (Palakodety, KhudaBukhsh, and Carbonell 2020), we fine-tune BERT on four sub-corpora: $\mathcal{D}^{old}_{bolly}$, $\mathcal{D}^{old}_{holly}$,$\mathcal{D}^{old}_{bolly}$, and $\mathcal{D}^{old}_{holly}$. We denote the pretrained BERT model as $\texttt{BERT}_{base}$ and a fine-tuned BERT on corpus $\mathcal{D}$ as $\texttt{BERT}_{\mathcal{D}}$. Out of a thorough analysis with several cloze tests with phrase variations, due to paucity of space, in Table 1 we report only three such results for the following cloze tests: (1) *A beautiful woman should have* [MASK] *skin*. (*cloze1*); (2) *A woman should be a* [MASK] *by occupation*. (*cloze2*); and (3) *A man should be a* [MASK] *by occupation* (*cloze3*).

Summary of findings: Given cultural and social bias toward fair skin prevalent in India for years (Karan 2008), it is not surprising that Bollywood exhibits an affinity toward fairness. However, our analysis indicates Hollywood also shows bias toward lighter skin color. Further, we observe strong presence of gender bias with masked sentences involving men predominantly receiving positive completions in comparison with women (see, Table 1). Future directions include (1) tracking moral sentiment; (2) exploring debiasing techniques; and (3) analyzing representation of other minorities.

References

Devlin, J.; Chang, M.-W.; Lee, K.; and Toutanova, K. 2019. BERT: Pre-training of Deep Bidirectional Transformers for Language Understanding. In *NAACL-HLT*, 4171–4186.

Garg, N.; Schiebinger, L.; Jurafsky, D.; and Zou, J. 2018. Word embeddings quantify 100 years of gender and ethnic stereotypes. *Proceedings of the National Academy of Sciences* 115(16): E3635–E3644.

Karan, K. 2008. Obsessions with fair skin: Color discourses in Indian advertising. *Advertising & society review* 9(2).

Khan, S.; and Taylor, L. 2018. Gender Policing in Mainstream Hindi Cinema: A Decade of Central Female Characters in Top-Grossing Bollywood Movies. *International Journal of Communication* 12: 22.

Lewis, M.; and Lupyan, G. 2020. Gender stereotypes are reflected in the distributional structure of 25 languages. *Nature human behaviour* 1–8.

Lison, P.; and Tiedemann, J. 2016. Opensubtitles2016: Extracting large parallel corpora from movie and tv subtitles .

Madaan, N.; Mehta, S.; Agrawaal, T.; Malhotra, V.; Aggarwal, A.; Gupta, Y.; and Saxena, M. 2018. Analyze, detect and remove gender stereotyping from bollywood movies. In *Conference on Fairness, Accountability and Transparency*, 92–105.

Palakodety, S.; KhudaBukhsh, A. R.; and Carbonell, J. G. 2020. Mining Insights from Large-Scale Corpora Using Fine-Tuned Language Models. In *ECAI 2020*, 1890–1897.

Petroni, F.; Rocktäschel, T.; Riedel, S.; Lewis, P.; Bakhtin, A.; Wu, Y.; and Miller, A. 2019. Language Models as Knowledge Bases? In *EMNLP-IJCNLP*, 2463–2473.

Taylor, W. L. 1953. "Cloze procedure": A new tool for measuring readability. *Journalism quarterly* 30(4): 415–433.

Twenge, J. M.; Campbell, W. K.; and Gentile, B. 2012. Male and female pronoun use in US books reflects women's status, 1900–2008. *Sex roles* 67(9-10): 488–493.
