# Supplementary material for: Gender bias, social bias, and representation: 70 years of BHollywood
Source: Patterns (N Y). 2021 Dec 9;3(2):100409. doi: 10.1016/j.patter.2021.100409 (PMC8848024; doi:10.1016/j.patter.2021.100409)
Supplement: Document S2. Supplemental experimental procedures and Tables S1–S5 [file mmc2.pdf]

Gender Bias, Social Bias and Representation: 70 Years of B^{H}ollywood

Kunal Khadilkar[a], Ashiqur R. KhudaBukhsh[b,*], Tom M. Mitchell[c]

[a]*School of Computer Science, Language Technologies Institute, Carnegie Mellon University, 5000 Forbes Avenue, Pittsburgh, PA, USA, 15213*
[b]*Golisano College of Computing and Information Sciences, Software Engineering Department, Rochester Institute of Technology, 20 Lomb Memorial Drive, New York, NY, USA, 14623*
[c]*School of Computer Science, Machine Learning Department, Language Technologies Institute, Carnegie Mellon University, 5000 Forbes Avenue, Pittsburgh, PA, USA, 15213*

1. Supplemental Information

1.1. MPR numerical values

Time Period	*Hollywood*	*Bollywood*
1950–1959	66.1 [64.3. 67.8]	58.0 [57.1, 58.9]
1960–1969	66.2 [64.9, 67.5]	58.2 [57.0, 59.3]
1970–1979	68.0 [66.6, 69.4]	62.4 [60.9, 63.9]
1980–1989	63.1 [61.3, 64.9]	64.4 [62.6, 66.2]
1990–1999	66.3 [64.8, 67.8]	62.1 [60.0, 64.2]
2000–2009	65.0 [63.7, 66.3]	61.7 [60.7, 62.7]
2010–2020	63.0 [61.2, 64.7]	61.5 [60.4, 62.6]

Table S.1: Numerical values for the *MPR* with confidence intervals. The confidence intervals are calculated using bootstrapping, with a 95% confidence level.

1.2. Implementation details

Experiments are conducted on a Google Colab Pro instance, using the Tesla V100 and P100 GPUs provided by Google in the Colab notebook.

1.3. Prominent Indian surnames

Table S.2 lists a random sample of surnames of three prominent Indian religions found in our data set.

*Corresponding author and the lead contact author
Email addresses: kkhadilk@cs.cmu.edu (Kunal Khadilkar), axkvse@rit.edu (Ashiqur R. KhudaBukhsh), tom.mitchell@cs.cmu.edu (Tom M. Mitchell)

[1]Kunal Khadilkar and Ashiqur R. KhudaBukhsh are equal contribution first authors.

Religion	Surnames
Hindu	Acharya, Sharma, Thakur, Mukherjee, Gupta
Muslim	Khan, Ali, Rehman, Akhtar, Kazmi
Sikh	Singh, Bedi, Chadha, Dhingra, Gill

Table S.2: Random samples of surnames for prominent Indian religions found in our data set.

1.4. WEAT results for different time periods

Table S.3 presents the WEAT scores for a different temporal binning and indicates that the qualitative claim of improvement in gender bias over time across both industries remains unchanged.

	1950 − 1969	*1970 − 1995*	*1996 − 2020*
Bollywood	0.601 [0.572, 0.630]	0.531 [0.504, 0.557]	0.518 [0.496, 0.539]
Hollywood	0.456 [0.441, 0.471]	0.427 [0.383, 0.470]	0.398 [0.360, 0.435]

Table S.3: WEAT scores for Bollywood and Hollywood across different time periods. Each cell summarizes the WEAT score for a given corpus as a $[b, c]$ where a denotes the WEAT score averaged over five runs (a larger positive value indicates greater bias toward men) and $[b, c]$ denotes the confidence interval with a 95% confidence level.

1.5. Additional cloze test results

Tables S.4 and S.5 present additional cloze test results for the time period 1970–1999. Tables 4 shows that for both men and women, the valence scores for both Bollywood and Hollywood show similar increasing trends over time. Table 5 show that across all time periods, both in Bollywood and Hollywood, all fine-tuned models output `fair` as the top completion.

**Probe**	$\texttt{BERT}_{\mathcal{D}^{mid}_{bolly}}$	$\texttt{BERT}_{\mathcal{D}^{mid}_{holly}}$
$cloze_1$	woman (0.094), servant (0.081), prostitute (0.078), man (0.077), slave (0.073), mother(0.071), worker(0.067), wife(0.056), doctor (0.052), lawyer(0.051), widow (0.051) [**4.9**]	woman (0.078), nurse (0.076), man (0.073), teacher (0.059), person (0.057), lawyer (0.056), doctor (0.039), worker (0.034), secretary (0.021), prostitute(0.014), lady (0.009) [**5.6**]
$cloze_2$	man (0.081), servant (0.081), lawyer (0.072), policeman (0.061), worker (0.049), gentleman (0.043), bachelor (0.041), doctor (0.028), criminal (0.024), farmer (0.016), businessman (0.009) [**5.1**]	man (0.088), soldier (0.072), policeman (0.059), gentleman (0.043), lawyer (0.032), doctor (0.031), bachelor (0.019), farmer (0.011), worker (0.009), teacher (0.007), minister (0.002) [**5.7**]

Table S.4: Cloze test results. Predicted tokens are ranked by decreasing probability. $\texttt{BERT}_{\mathcal{D}}$ denotes $\texttt{BERT}$ trained on corpus $\mathcal{D}$. $\mathcal{D}^{mid}_{bolly}$ and $\mathcal{D}^{mid}_{holly}$ consist of movies between 1970-1999 in our Bollywood and Hollywood data set, respectively. The number in the bracket represents the average valence score (computed using a well-known lexicon presented in[1]) calculated for the cloze test outputs. The $\texttt{BERT}$ probabilities are mentioned in parentheses.

$\texttt{BERT}_{\mathcal{D}^{mid}_{bolly}}$	$\texttt{BERT}_{\mathcal{D}^{mid}_{holly}}$
fair (0.067), tanned (0.045), dark (0.041), white (0.025), beautiful (0.021)	fair (0.073), pale (0.054), tanned (0.043), no (0.042), golden (0.033)

Table S. 5: Cloze test results for the probe *A beautiful woman should have* `[MASK]` *skin*. Predicted tokens are ranked by decreasing probability with probabilities mentioned in parentheses. $\texttt{BERT}_{base}$ denotes the pre-trained $\texttt{BERT}$. $\texttt{BERT}_{\mathcal{D}}$ denotes $\texttt{BERT}$ fine-tuned on corpus $\mathcal{D}$. $\mathcal{D}^{mid}_{holly}$ and $\mathcal{D}^{mid}_{bolly}$ consist of movies between 1970-1999 in our Hollywood and Bollywood data set, respectively.

References

[1] A. B. Warriner, V. Kuperman, M. Brysbaert, Norms of valence, arousal, and dominance for 13,915 English lemmas, Behavior research methods 45 (4) (2013) 1191–1207.
