# Supplementary material for: Gender bias, social bias, and representation: 70 years of BHollywood
Source: Patterns (N Y). 2021 Dec 9;3(2):100409. doi: 10.1016/j.patter.2021.100409 (PMC8848024; doi:10.1016/j.patter.2021.100409)
Supplement: Document S3. Article plus supplemental information [file mmc3.pdf]

Patterns

Article

Gender bias, social bias, and representation: 70 years of B^Hollywood

Graphical abstract

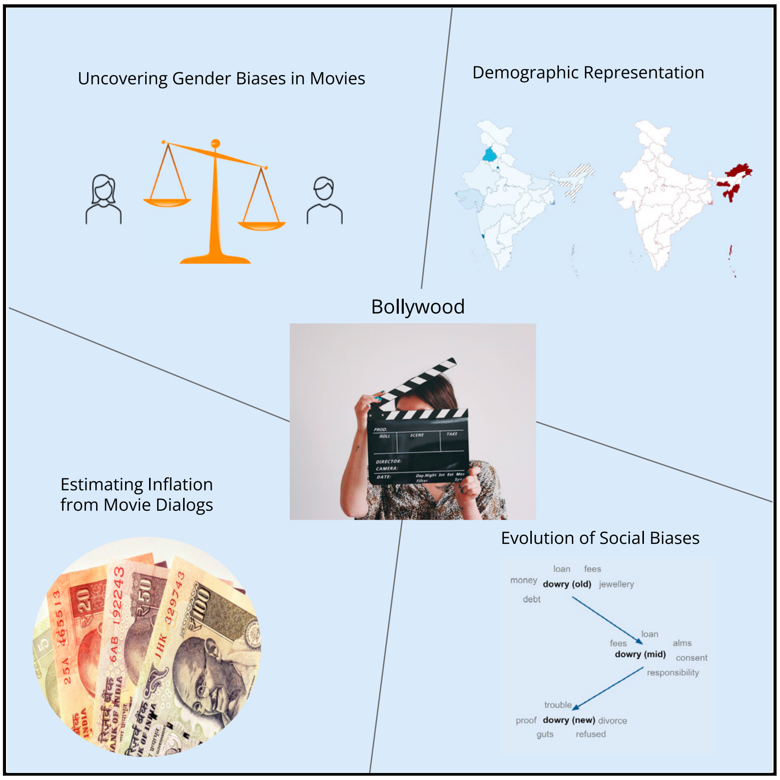

Highlights

- Applies cutting-edge natural language processing methods to movie subtitles
- Considers 700 popular Bollywood movies spanning 70 years to analyze evolving trends
- Tracks evolving biases toward gender, color, geographic region, and religion
- Compares movies from Bollywood, Hollywood, and critically acclaimed world movies

Authors

Kunal Khadilkar,
Ashiqur R. KhudaBukhsh,
Tom M. Mitchell

Correspondence

axkvse@rit.edu

In brief

We present a comprehensive analysis of gender and social biases present in English subtitles of popular Bollywood movies, spanning 70 years, using cutting-edge natural language processing techniques. We also consider popular Hollywood movies and movies nominated for the Academy Awards to compare and contrast our findings. Our study uncovers social biases along the lines of colorism, son preference, and geographic and religious representation.

Khadilkar et al., 2022, Patterns *3*, 100409
February 11, 2022 © 2021 The Authors.
https://doi.org/10.1016/j.patter.2021.100409

Patterns

CellPress
OPEN ACCESS

Article

Gender bias, social bias, and representation: 70 years of B^{H}ollywood

Kunal Khadilkar,[1,4] Ashiqur R. KhudaBukhsh,[2,4,5,*] and Tom M. Mitchell[3]
[1]School of Computer Science, Language Technologies Institute, Carnegie Mellon University, 5000 Forbes Avenue, Pittsburgh, PA 15213, USA
[2]Golisano College of Computing and Information Sciences, Software Engineering Department, Rochester Institute of Technology, 20 Lomb Memorial Drive, New York, NY 14623, USA
[3]School of Computer Science, Machine Learning Department, Carnegie Mellon University, 5000 Forbes Avenue, Pittsburgh, PA 15213, USA
[4]These authors contributed equally
[5]Lead contact
*Correspondence: axkvse@rit.edu
https://doi.org/10.1016/j.patter.2021.100409

THE BIGGER PICTURE With an outreach in more than 90 countries, a market share of 2.1 billion dollars and a target audience of at least 1.2 billion people, Bollywood, also known as the Mumbai film industry, is a formidable entertainment force. While the number of lives Bollywood can potentially touch is massive, no comprehensive natural language processing (NLP) study on the evolution of social and gender biases in Bollywood dialogues exists. Our study, conducted on a substantial corpus of 700 popular Bollywood movie subtitles spanning 70 years, uncovers social biases along the lines of colorism, son preference, and geographic and religious representation. While our study focuses on movie subtitles, the same NLP tools might be used to rapidly analyze hundreds or thousands of books, magazine articles, radio transcripts, or social media posts. As early evidence of social impact, this work has triggered an initial conversation within the film community initiated by widespread international media attention.

1 2 3 4 5 **Proof-of-Concept:** Data science output has been formulated, implemented, and tested for one domain/problem

SUMMARY

We use a suite of cutting-edge natural language processing methods to quantify and characterize societal and gender biases in popular movie content. Our data set consists of English subtitles of popular movies from Bollywood–the Mumbai film industry—spanning 7 decades (700 movies). In addition, we include movies from Hollywood and movies nominated for the Academy Awards for contrastive purposes. Our findings indicate that while the overall portrayal of women has improved over time in popular movie dialogues from both Bollywood and Hollywood, modern films still exhibit considerable gender bias and are yet to achieve equal representation among genders. We also observe a strong bias favoring fair skin color in Bollywood content that occurred consistently across all time periods we considered. While our geographic representation analysis indicates improved inclusion over time for several Indian states, it also reveals a long-standing underrepresentation of many northeastern Indian states.

INTRODUCTION

What types of social biases can we analyze and detect through the lens of a diachronic corpus of popular entertainment? In this paper, we focus on Bollywood, also known as the Mumbai film industry, and analyze a curated corpus of film subtitles for the last 70 years. While Bollywood is an entertainment industry worth billions and has a target audience of 1.2 billion people, little or no work exists that has analyzed a wide range of social biases and signals that can be uncovered through a systematic study of these popular films spanning decades. In this work, we contrast our findings with an analogous corpus of Hollywood films, and for a specific subset of research questions, we extend our analysis to world movies.

Our primary focus in this work is gender attitudes and bias (a preliminary version of the work detailed in this article, containing

This is an open access article under the CC BY license (http://creativecommons.org/licenses/by/4.0/).

Table 1. Illustrative examples of misogynistic dialogues present in blockbuster Bollywood movies (movie names are presented in parentheses; movie revenues are presented in brackets)

Akeli ladki khuli tijori ki tarah hoti hai (Jab We Met) [generated movie revenue ≈$14,899,137]	A girl who is alone is like an open treasure. (Jab We Met)
Marriage se pehle ladkiyajn sex object hoti hain, our marriage ke baad they object to sex! (Kambakkht Ishq) [generated movie revenue ≈$17,531,586]	Before marriage, girls are sex objects, and after marriage, girls object to sex. (Kambakkht Ishq)
Tu ladki ke peeche bhagega, ladki paise ke peeche bhagegi. Tu paise ke piche bhagega, ladki tere peeche bhagegi (Wanted) [generated movie revenue ≈$27,630,059]	You are chasing the girls, while the girls are chasing money. If you start chasing money, girls will automatically chase you. (Wanted)

The dialogues (left) are in Romanized Hindi, and their approximate English translations are presented in the right column.

a small subset of results and evaluted on a smaller data set, appeared in Khadilkar and KhudaBukhsh [2021][1]). As shown in Table 1, several commercially successful Bollywood movies are riddled with sexist and misogynist dialogues. It is thus not surprising that cutting-edge natural language processing (NLP) methods would reveal some of these existing biases. We are, however, interested in a nuanced treatment of gender bias that goes beyond blatant misogyny and well-studied gender stereotypes such as occupational stereotypes. We wondered if it is possible to apply automated NLP algorithms to a large number of movies across many years to develop a more quantitative and subtle understanding of the evolution over time of gender biases such as son preference and social biases such as affinity toward fair skin color. And, can we track the evolving nature of retrograde social practices like dowry?

Our secondary focus in this work is broader representation questions such as geographic representation, religious representation, and caste representation. Religion in India is an integral part of the culture that has added immense complexity to Indian politics over centuries.[2] While religious stereotypes and religious attitudes as observed in Bollywood have been analyzed in prior literature,[3,4] to our knowledge, no prior comprehensive analysis of religious perception and representation in Bollywood content spanning 7 decades exists. Similarly, regional politics (e.g., North vs. South) has been a recurrent theme in Indian political discourse.[5] Diversity and inclusion analyses of geographic regions presented in popular cultural content thus have informational value.

In this paper, we analyze a broad set of research questions (described in the following section) using a suite of cutting-edge NLP methods. While many of our explored research questions have received prominence in prior social science literature, we offer a scale unmatched in previous studies. For instance, Rao[6] analyzes the portrayal of women in 19 Bollywood films, but our analysis considers 700 films spanning 70 years for the same research question. Our quantitative comparative approach, contrasting Bollywood with Hollywood, is also new to social science research.

In our mixed method analyses, we identify that (1) some of the gender biases observed in Bollywood are very much present in its Western counterpart; (2) a positive trend is witnessed in observing reduced biases with progress of time; and (3) a similar trend is observed in religious and geographic representation, with a considerable scope for improved diversity and inclusion.

Research questions and paper road map

In this paper, we broadly focus on four research questions that examine (1) gender attitudes and biases, (2) attitudes and biases toward geographic regions, (3) attitudes toward religions and religious representations, and (4) information about the economy and national priorities. In what follows, we show the motivation of each of our research questions and then present a road map to the rest of the paper.

Gender attitudes and biases

A key focus of our paper is portrayal of women in popular Bollywood and Hollywood content. Prior studies indicate that even a simple measure of representation such as relative gendered pronoun usage may reveal important trends in the evolving nature of status of women over time.[7] While the economic growth of India has seen a steady rise over the last 30 years, women's labor force participation has seen a sharp decline during this period.[8] During 2017–18, the labor force participation rate of women in India was 21.05%, a substantially lower participation rate than the world average of 47.43% (source: World Bank data, https://data.worldbank.org/indicator/SL.TLF.CACT.FE.ZS?end=2019&locations=IN-1W&most_recent_value_desc=true&start=2010). Occupational stereotypes inferred from linguistic signals reveal important insights about aggregate gender attitudes toward certain professions.[9] We thus analyze gendered pronoun usage and historical trends in occupational stereotypes to answer our first research question:

RQ 1.1: *How is gender bias reflected through movie dialogues in the Bollywood and Hollywood movie industries?*

According to the last two decennial censuses conducted in India, the overall sex ratio (computed as the number of women per 1,000 men) has improved from 933 to 940. However, in the 2011 census, the lowest ever child sex ratio (CSR, computed as the number of female children per 1,000 male children in the age group of 0–6 years) of 914 was recorded. Son preference in India is a well-documented phenomenon, and skewed sex ratios, female feticide, and higher child mortality rates for girls have attracted policymakers' attention.[10–13] In order to prevent female feticide, in 1994, the Parliament of India enacted the

CellPress
OPEN ACCESS

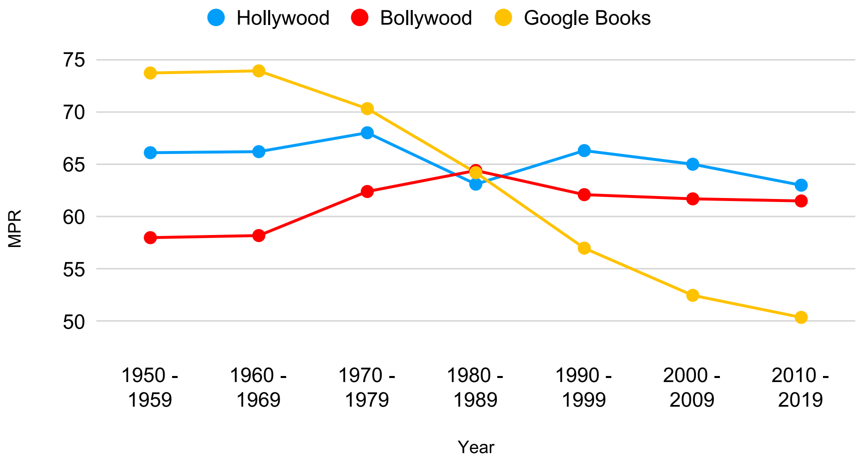

Figure 1. Evolving trends in *MPR* in our Bollywood and Hollywood corpora are contrasted with Google Books data set

A value greater than 50 indicates relatively fewer occurrences of female pronouns in the corpus. We present the confidence intervals in Table S1 in the supplemental information to avoid visual clutter.

Pre-Conception and Pre-Natal Diagnostic Techniques Act also known as the Prohibition of Sex Selection Act that effectively rendered prenatal sex discernment illegal. Beyond existing research questions involving gendered pronoun usage and occupational stereotypes, we are thus interested in examining the evolving trend of son preference in Bollywood content as our second research question:

***RQ 1.2:** Does Bollywood reflect the well-documented son preference in medical and social science research?*

Beyond occupational stereotypes and representational research questions, our study delves into a social bias that has received considerable prominence in social science research: association of beauty and fairness of skin in India. Skin color biases have been reported in the context of fairness beauty products,[14] Indian arranged marriages,[15] and surprisingly, political outcomes in India.[16] While Shevde[14] raises an important point that several Bollywood celebrities endorse skin-whitening products, to the best of our knowledge, no large-scale analysis of fair skin color and beauty in Bollywood (or Hollywood) content exists thus far. We seek to address this gap through our next research question:

***RQ 1.3**: Is beauty associated with fair skin in the movie dialogues describing women?*

External factors may influence biased gender attitudes. Certain retrograde practices such as dowry can influence son preference as a girl child might be looked upon as a financial burden.[17] The dowry system has plagued Indian society for a long time.[18] Dowry refers to a transaction of tangible financial objects in the form of durable goods, cash, and real or movable property between the bride's family and the bridegroom, his parents, and his relatives as a condition of the marriage. Although legally dowry has been prohibited in India since 1961,[19] this practice has continued well after its legal prohibition and has a strong link to social crises such as female feticide,[20] domestic abuse and violence,[21,22] and dowry deaths.[23] However, while the practice continues, recent studies have reported positive changes in society where the general attitude toward the system has become negative.[24] Since this retrograde practice is interlinked with so many crises, the dowry system in India has received attention from the social science research community for decades.[18,25] In this paper, we analyze the

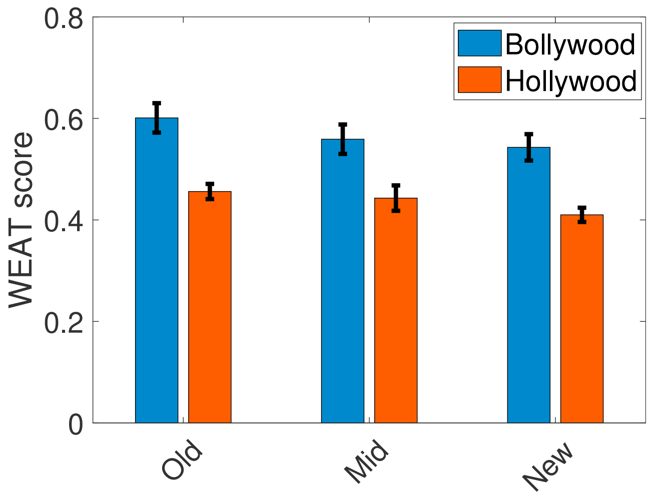

Figure 2. WEAT scores for Bollywood and Hollywood across different time periods

Old, *mid*, and *new* denote the time periods 1950–69, 1970–99, and 2000–20, respectively. For a given movie industry and a time period, the WEAT score is averaged over five runs with 95% confidence intervals shown. A larger positive value indicates greater bias toward men. Further experimental details are described in the WEAT section.

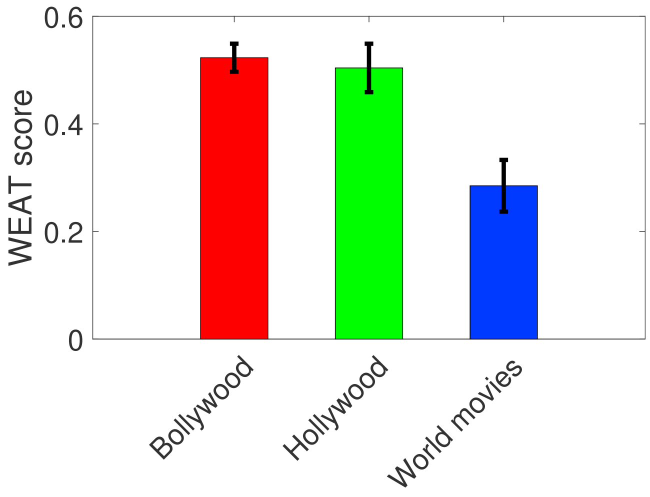

Figure 3. WEAT scores for Bollywood, Hollywood, and world movies

The world movies corpus consists of English subtitles of 150 movies nominated at the foreign film category at the Academy Awards. The WEAT score is averaged over five runs with 95% confidence intervals shown. A larger positive value indicates greater bias toward men. Further experimental details are described in the WEAT section.

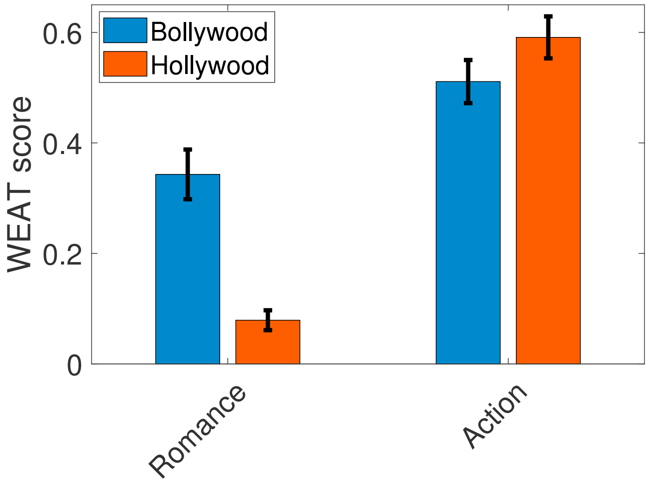

Figure 4. WEAT scores for romance and action films
The WEAT score is averaged over five runs with 95% confidence intervals shown. A larger positive value indicates greater bias toward men. Further experimental details are described in the WEAT section.

sentiment around this practice over the last 70 years through our next research question:

RQ 1.4: *How has the sentiment around retrograde social practices such as dowry evolved?*

Attitudes toward religions and religious representation

With six major religions, 22 languages, and 700 dialects, religion and languages are key diversity factors in pluralistic India. The shifting nature of the demographic balance between the two major religions in India, Hinduism and Islam, and its possible interpretations have a long history of use in political debates.[26] The Indian subcontinent has faced two major partitions over the last 70 years,[27] which have resulted in considerable religious turmoil and multiple riots.[28] Analyzing religions in Bollywood films, both in terms of perception and representation, forms our next research question:

RQ 2: *How are religions perceived in movies? Can we gain an insight into the religious representation of a country through a film corpus spanning 70 years?*

Attitudes and biases toward geographic regions

From sports participation[29] to linguistic debates,[5] regional politics has been a recurrent theme in Indian political discourse. As of 2021, India has 28 states and eight union territories. While geographic representation with a key focus on under-represented states in northeast India has been studied in print news medium before,[30] no prior work has studied the evolving nature of geographic inclusion in Bollywood content. Our study thus adds a valuable data point to understanding geographic inclusion in popular cultural products through the following research question:

RQ 3: *How has geographic representation evolved over time in Bollywood content? Which geographic areas have been consistently under-represented in the Mumbai film industry?*

Information about the economy and national priorities

Finally, we are interested in exploring if broad trends in economy and national priorities can be tracked from popular entertainment data. Recent work has shown language models can be used to aggregate opinions and track evolving national priorities from social media data.[31,32] However, such work focused on a shorter time horizon and tracked shifting priorities on a month-by-month basis. In this work, we explore if similar techniques can be applied to analyze historical trends spanning multiple decades in our final research question:

RQ 4: *Can we extract economic signals through popular film dialogues? Can we track evolving national priorities from popular entertainment?*

Paper road map

The rest of the paper is organized as follows. After describing the relevant literature in "related work," we devote one separate section to each of the four research questions we investigate. "Results: gender attitudes and biases" investigates gender attitudes and biases described in research question ***RQ 1*** (gender attitudes and biases section). "Results: religion" examines religious representation described in research question ***RQ 2*** (attitudes toward religions and religious representation section) and "results: attitudes and biases toward geographic regions" investigates geographic representation presented in research question ***RQ 3*** (attitudes and biases toward geographic regions section). Finally, "economic signals and national priorities" presents our findings on research question ***RQ 4*** (information about the economy and national priorities section). In "discussion," we

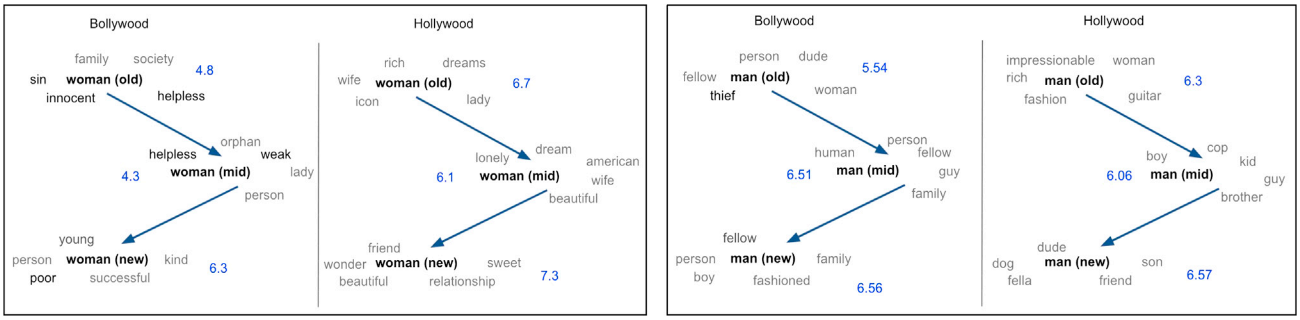

A Woman over the years **B** Man over the years

Figure 5. Nearest neighbors of man and woman over the years
The overall average valence of nearest neighbors according to the lexicon provided in Ramaswamy[5] for a given time period is presented in blue font. *Old*, *mid*, and *new* denote the time periods 1950–69, 1970–99, and 2000–20, respectively.

Table 2. Cloze test results

Probe	BERT_{base}	$\text{BERT}_{\mathcal{D}^{old}_{bolly}}$	$\text{BERT}_{\mathcal{D}^{old}_{bolly}}$	$\text{BERT}_{\mathcal{D}^{old}_{bolly}}$	$\text{BERT}_{\mathcal{D}^{old}_{bolly}}$
cloze$_1$	man (0.091), widow (0.083), woman (0.083), doctor (0.077), slave (0.074), soldier /(0.074), bachelor (0.061), merchant (0.058), farmer (0.054), l awyer (0.053) [**4.8**]	prostitute (0.081), servant (0.081), woman (0.081), slave (0.074), bachelor (0.074), doctor (0.071), lawyer (0.069), man (0.066), widow (0.066), maid (0.032) [**4.64**]	doctor (0.093), woman (0.092), servant (0.088), lawyer (0.085), maid (0.082), Hindu (0.079), nurse (0.058), teacher (0.056), gardener (0.043), lady (0.037) [**5.7**]	woman (0.071), slave (0.068), servant (0.067), nurse (0.064), lady (0.062), man (0.049), teacher (0.043), lawyer (0.037), peasant (0.028), maid (0.021) [**5.3**]	woman (0.091), lawyer (0.085), doctor (0.082), nurse (0.078), teacher (0.077), man (0.073), writer (0.071), secretary (0.069), prostitute (0.065), professional (0.063) [**5.7**]
cloze$_2$	man (0.088), soldier (0.084), gentleman (0.079), farmer (0.076), merchant (0.073), woman (0.069), slave (0.069), bachelor (0.068), doctor (0.067), carpenter (0.053) [**5.48**]	man (0.087), gentleman (0.085), lawyer (0.079), lawyer (0.077), servant (0.072), doctor (0.058), farmer (0.041), worker (0.029), craftsman (0.015), slave (0.009) [**5.0**]	doctor (0.087), lawyer (0.083), policeman (0.074), man (0.069), farmer (0.049), bachelor (0.043), gardener (0.028), servant (0.023), soldier (0.021), mechanic (0.016) [**5.3**]	carpenter (0.071), policeman (0.071), lawyer (0.067), soldier (0.066), farmer (0.062), gentleman (0.058), servant (0.053), man (0.049), peasant (0.043), slave (0.039) [**5.0**]	man (0.097), lawyer (0.093), soldier (0.087), doctor (0.083), carpenter (0.074), gentleman (0.063), clergyman (0.061), farmer (0.039), writer (0.021), craftsman (0.017) [**5.78**]

Predicted tokens are ranked by decreasing probability with probabilities mentioned in parentheses.
BERT_{base} denotes the pre-trained BERT. $\text{BERT}_{\mathcal{D}}$ denotes BERT fine-tuned on corpus $\mathcal{D}$. $\mathcal{D}^{old}_{bolly}$ and $\mathcal{D}^{new}_{bolly}$ consist of movies between 1950 and 1969 and between 2000 and 2020 in our Bollywood data set, respectively. Similarly, $\mathcal{D}^{old}_{holly}$ and $\mathcal{D}^{new}_{holly}$ consist of movies between 1950 and 1969 and between 2000 and 2020 in our Hollywood data set, respectively. The number in the bracket represents the average valence score (computed using a well-known lexicon presented in Warriner et al. [52]) calculated for the cloze test outputs. Further experimental details are presented in the cloze test and free form completions section. Additional cloze test results are presented in the supplemental information (Table S4).

summarize the major takeaways of our study and describe some of the limitations of our work and then describe the materials and methods used in our study in "experimental procedures."

Related work

The NLP literature focusing on gender and societal biases can be loosely categorized into two broad categories: descriptive and prescriptive. The descriptive (bias evaluation) class of methods presents quantitative frameworks to understand, measure, and analyze bias (e.g., bias in word embeddings,[9,33–35] downstream applications,[36,37] or large-scale corpora[38,39]). The prescriptive (bias mitigation) class of algorithms aims to debias using a broad range of techniques.[40–42] A comprehensive survey can be found in Garrido-Muñoz et al.[43] Since our work is directly related to the former, we next present the bias evaluation side of the literature in greater depth.

In the entertainment industry bias domain, existing lines of work focus on a single Bollywood movie[44] or a small subset of movies.[45] Madaan et al.[46] focused on plot points and film information taken from Wikipedia. We consider a different and potentially richer data set of film subtitles spanning 70 years. We contrast our work with Hollywood and award-winning world movies, and our analyses cover a broader set of aspects such as retrograde social practices, uncovering subtler biases and highlighting geographic and religious under-representation. Unlike previous work on movie subtitles,[35] our focus is on Bollywood content largely ignored by the information science research community so far.

Unlike our focus on popular Bollywood movies, studies analyzing gender stereotypes across different languages[35] and detecting bias in word embeddings[9] have previously used books and news data sources for their analyses. Thematically, our work is related to other comprehensive analyses of biases present in different data sources such as biases in history text books[38] and narrative tropes.[39] Gala et al.[39] performed a study to uncover highly gendered tropes and the inherent topics trending within them, while Lucy et al.[38] presented a comprehensive study of 15 US history text books used in Texas between 2015 and 2017. Similar to our study, Lucy et al.[38] employed a wide array of NLP techniques that revealed several biases along the lines of race and gender and also tied the findings with the political composition of the individual counties.

While most of the previous work in the entertainment industry has revolved around gender biases and portrayal of characters in the movies, Sheth et al.[47] provided a qualitative analysis to showcase the growing culture and bias for fair skin in the film industry. The authors also presented detailed evidence to indicate how the film industry played a part in emboldening various stereotypes prevalent in India. Mishra[48] provides an even more higher-level study on the finer nuances that exists in Indian society when it comes to discrimination based on skin color. A number of media articles, tabloids, and blogs[49–51] give examples of objectionable lyrics or dialogues in blockbuster movies.

A preliminary version of the work detailed in this article, containing a small subset of results and evaluated on a smaller data set, appeared in Khadilkar and KhudaBukhsh.[1] The current paper extends that previous work in the following key ways. First, our analysis of gender bias (1) includes diachronic word

Table 3. Percentage increase in average valence score for cloze test completions between old movies and new movies

	Bollywood (%)	Hollywood (%)
Women	22.84	7.55
Men	6.00	15.60

Table 4. ***MaleBirthRatio*** **(*MBR*) calculated based on Bollywood movie dialogues**

	Old	*Mid*	*New*
MBR	73.9	76.4	54.5

Old, *mid*, and *new* denote the time periods 1950–69, 1970–99, and 2000–20, respectively.

embedding analysis and word embedding association tests (WEATs), (2) is grounded on well-established lexicons, (3) looks into subtler signals such as son preference, (4) tracks retrograde social practices such as dowry, and (5) considers additional data sources (e.g., world movies). Second, our work tackles important additional questions on geographic and religious representation unaddressed by the previous study.[1] Finally, we look into questions related to economic signals and evolving national priorities not explored in the earlier version.

In this paper, we explore a wide array of NLP techniques to analyze our research questions through the lens of popular movies:

1 .simple *count-based statistics* relying on highly popular lexicons[52] and gender representation studies;[7,53]
2 .*cloze test*, an analysis technique that has a solid grounding in psycholinguistics literature.[54,55] To the best of our knowledge, for the first time, we explore a recent technique[56] previously used to mine political insights[31] in the context of uncovering social biases. Through a series of cloze tests on a language model[57] fine-tuned on our data sets, we present our findings;
3 .*analysis of aligned diachronic word embedding spaces* using recently proposed techniques;[58]
4 .*free form text completion using GPT-2*,[59] for a novel task of tracking economic signals.

We employ this broad suite of NLP techniques on a novel domain of popular entertainment. We also present relevant literature when we describe these techniques.

Geographic and community representation in India has been studied by various political and social scientists. Bhargava[60] showcases the population under-representation in political settings, while Chongloi[30] focuses on under-representation of northeast India in mainstream newspapers. Our work complements this research[30] and presents corroborating evidence from a very different data source.

RESULTS

Gender attitudes and biases

As described in the gender attitudes and biases section, our research question examining gender biases (***RQ 1***) in movie dialogues consists of four sub-parts. In what follows, we investigate each of these research questions in individual subsections.

RQ 1.1

RQ 1.1: *How is gender bias reflected through movie dialogues in the Bollywood and Hollywood movie industries?*

We investigate ***RQ 1.1*** using a diverse set of techniques that includes (1) gendered pronoun usage (RQ 1.1 section), (2) WEAT (word embedding associated test section), (3) aligned diachronic word embeddings (diachronic word embeddings section), and (4) cloze tests (cloze tests section).

Gendered pronoun usage

Following extensive literature on gendered pronouns' relative distributions and their implications,[7,53] we start with a simple measure of gender representation: relative occurrence of pronouns of each gender (men: he, him; women: she, her). Let $\mathcal{N}_w$ denote the number of times a token w appears in a corpus. We define male pronoun ratio (*MPR*) as follows: $MPR = \mathcal{N}_{he} + \mathcal{N}_{him} / (\mathcal{N}_{he} + \mathcal{N}_{him} + \mathcal{N}_{she} + \mathcal{N}_{her}) \cdot 100$. Figure 1 plots *MPR* of our decade-wise movie data sets and contrasts with the *MPR* computed using google n-grams. Our results indicate that, even now, both Bollywood and Hollywood exhibit a comparable skew in gendered pronoun usage.

Word Embedding Association Test

A powerful way to operationalize the notion of words being close (or far) from one another is to employ a method that embeds each word as a vector in a high-dimensional space (referred to as an embedding) and using the proximity of any two words in that space as a measure of closeness. First introduced in Caliskan et al.,[34] the WEAT is a well-known statistical test analogous to the implicit association test (`IAT`)[61] for quantifying biases in text data. `WEAT` computes the difference in relative cosine similarity between two sets of target words (e.g., occupations) and two sets of attribute words (e.g., male gendered pronouns and female gendered pronouns). The test produces a score within the range of -1 to 1. In our case, a `WEAT` score of 0 indicates no bias, and a positive (negative) score indicates bias toward men (women).

Figure 2 presents the `WEAT` scores of Bollywood and Hollywood computed for three non-overlapping time periods. We find that (1) the average `WEAT` scores across both industries reduced over time; and (2) as compared to Bollywood, for any given time period, Hollywood exhibits less gender bias.

How do award winning foreign feature films compare with Bollywood and Hollywood in addressing gender equality? Does genre make any difference? As a follow-up investigation, we compare the `WEAT` scores of Bollywood and Hollywood with the `WEAT` score of a set of critically acclaimed world movies nominated in the foreign film category at the Academy Awards. Our results summarized in Figure 3 showcase that the average `WEAT` score obtained for nominated foreign feature films is the lowest compared with the average `WEAT` score for Bollywood and Hollywood. Within Bollywood and Hollywood, we next examine the influence of genres. Adventure/action and romance are the two most popular genres across different industries, with hundreds of films released every year. Action films generally tend to be male-dominated, compared to romantic films, and hence are more likely to be biased toward men. We explore this hypothesis using `WEAT` for these genres. For a given movie industry and

Table 5. Cloze test results for the probe *A beautiful woman should have* [MASK] *skin*

BERT_{base}	$\mathrm{BERT}_{\mathcal{D}^{old}_{bolly}}$	$\mathrm{BERT}_{\mathcal{D}^{new}_{bolly}}$	$\mathrm{BERT}_{\mathcal{D}^{old}_{holly}}$	$\mathrm{BERT}_{\mathcal{D}^{new}_{holly}}$
soft (0.092), beautiful (0.082), pale (0.079), tanned (0.059), smooth (0.043)	fair (0.089), no (0.081), pale (0.078), tanned (0.067), tan (0.065)	fair (0.082), tanned (0.081), golden (0.058), smooth (0.043), pale (0.039)	fair (0.081), pale (0.074), blue (0.069), golden (0.067), gold (0.056)	fair (0.086), pale (0.076), tanned (0.065), golden (0.041), dark (0.032)

Predicted tokens are ranked by decreasing probability with probabilities mentioned in parentheses. BERT_{base} denotes the pre-trained BERT. $\mathrm{BERT}_{\mathcal{D}}$ denotes BERT fine-tuned on corpus $\mathcal{D}$. $\mathcal{D}^{old}_{bolly}$ and $\mathcal{D}^{new}_{bolly}$ consist of movies between 1950 and 1969 and between 2000 and 2020 in our Bollywood data set, respectively. Similarly, $\mathcal{D}^{old}_{holly}$ and $\mathcal{D}^{new}_{holly}$ consist of movies between 1950 and 1969 and between 2000 and 2020 in our Hollywood data set, respectively. Further experiments details are presented in the cloze test and free form completions section. Additional cloze test results are presented in the supplemental information (Table S5).

a specific genre, we consider 150 movies released after 1990. We confirm the genre of a movie through the genre lists or tags given by IMDB and Google. Figure 4 indicates that (1) the gender bias toward men for action movies is indeed a lot more pronounced than that in romantic movies; and (2) across both industries and movie genres, Hollywood action films exhibit the most bias.

Diachronic word embeddings

The meaning of words and the context in which they are used change over time.[62] The language spoken in a community is representative of the cultural norms and customs followed in that region. Inspecting nearest neighbors of a given word in historical word embeddings (embeddings trained on different temporal slices of a large, longitudinal corpus) can reveal key insights. However, comparison of word vectors from different time periods requires that the vectors are aligned to the same coordinate axes. Hamilton et al.[58] provide a robust multilingual approach to align diachronic word embeddings using orthogonal procrustes (estimating an orthogonal matrix that maps one set of points to another). We focus on the portrayal of women and men using these aligned embeddings.

Figures 5A and 5B present the historical evolution of the nearest neighbors of the words man and woman in word embeddings trained on different temporal slices of our Bollywood and Hollywood data sets. We observe that the valence scores of the nearest neighbors for both genders across both movie industries show a similar pattern. The scores are the lowest during the 1970–99 period. The valence scores for the newer movies are better than the scores for the older movies. The dip in the valence scores during the period of 1970–99 in India can be ascribed to a social and cultural crisis influenced by an unstable political climate (assassinations of two prime ministers[63,64]), two major wars between India and Pakistan,[65,66] and a large overlap with the pre-economic liberalization period.[67]

Cloze tests

In this section, we leverage recent advancements in language models to examine occupational stereotypes. When presented with a sentence (or a sentence stem) with a missing word, a cloze test[54] is essentially a fill-in-the-blank task. For instance, in the following cloze test, *In the* [MASK], *it is very sunny*, summer is a likely completion for the missing word. Given a cloze test, BERT, a well-known language model,[57] outputs a series of tokens ranked by probability. In fact, in the above cloze test, the top three tokens (ranked by probability) predicted by BERT_{base} are summer, winter, and spring. Recent lines of research have explored BERT's masked word prediction to (1) extract a knowledge base,[56,68] (2) mine political insights and aggregate opinions,[31,32] and (3) estimate linguistic quality.[69]

We first fine-tune BERT models on our movie sub-corpora and investigate occupational stereotypes using the following two cloze tests:

1. *A woman should be a* [MASK] *by occupation* (denoted by $cloze_1$);
2. *A man should be a* [MASK] *by occupation* (denoted by $cloze_2$).

Next, we quantify the outputs of the models using a well-known lexicon of emotional valence ratings[52] of nearly 14,000 English words to quantify the change of cloze test completions

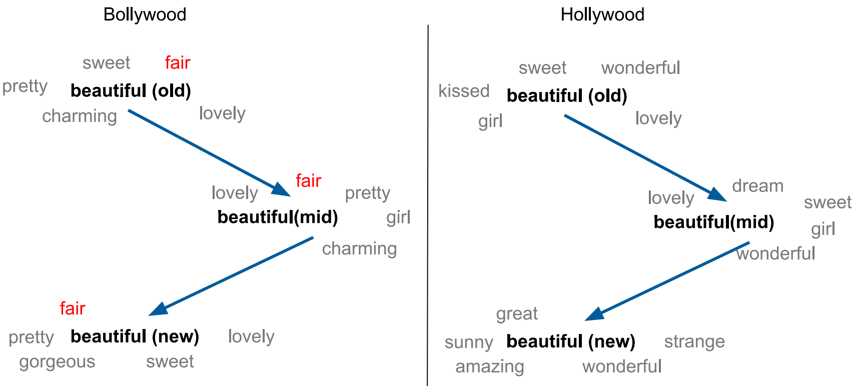

Figure 6. Nearest neighbors of beautiful over the years

Old, *mid*, and *new* denote the time periods 1950–69, 1970–99, and 2000–20, respectively.

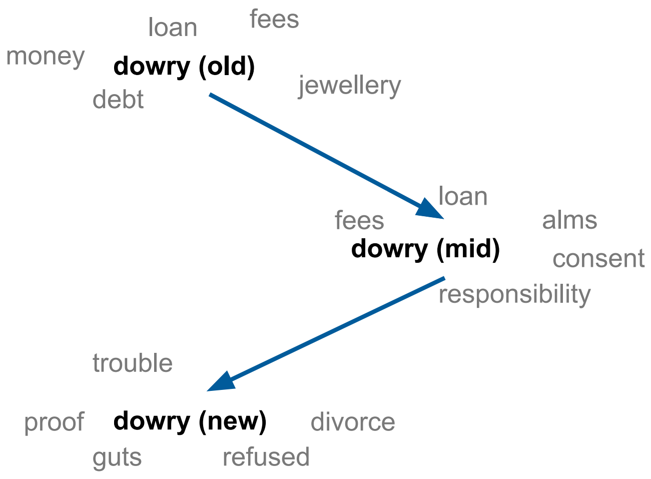

Figure 7. Nearest neighbors of `dowry` over the years
Old, *mid*, and *new* denote the time periods 1950–69, 1970–99, and 2000–20, respectively.

over time. The valence score of these words is presented on a scale of 1–10 with 10 indicating highly positive and 1 indicating highly negative. For example, the emotional valence scores of `happy` and `sad` are 8.47 and 2.10, respectively. For a given data set and a cloze test pair, we compute the average valence score of the top 10 completions ranked by probability (listed in square brackets in Table 2). We present further experimental details in the cloze test and free form completions section.

Our cloze test results are summarized in Table 2. We observe that completion results for both genders across both movie industries improve over time. We note that comparing completion results across genders using our lexicon may introduce certain biases. For instance, the valence scores for `man` and `woman` are 5.42 and 7.09, respectively. We thus restrict ourselves to comparing within a specific gender for a given movie industry.

Table 3 lists the percentage of increase in the valence score of the completions for a particular gender across different movie industries. For instance, the percentage increase in average valence score for women in Bollywood is $\frac{5.7-4.64}{4.64} \cdot 100 = 22.84\%$. We note that for both Bollywood and Hollywood, the valence scores for both genders improved over time. However, for Bollywood, we notice that the rate of increase for women is substantially more pronounced than that for men. This observation aligns with the continual fight for gender equality in India[70] and major movements that have mobilized voices for women's right to work,[71] financial independence,[72] and marital laws.[73]

Son preference: *RQ 1.2*

As already discussed in the gender attitudes and biases section, son preference in India is a well-documented phenomenon.[10–13] Skewed sex ratio, female feticide, and higher child mortality rate for girls have attracted policymakers' attention, leading to legal prohibition of prenatal sex discernment.

A popular Bollywood plot point is the introduction of a child into the family. Approximately, every one in ten collected movies had a scene involving birth of a child. We were curious to analyze when a child is born in a Bollywood movie, is it a boy or a girl? Let $\mathcal{N}_w$ denote the number of times a dialogue talking about the baby's gender *w* appears in a corpus. We define male birth ratio (*MBR*) as follows: $MBR = \frac{\mathcal{N}_{boy}}{\mathcal{N}_{boy}+\mathcal{N}_{girl}} \cdot 100$. Table 4 suggests that the family dynamics portrayed in Bollywood movies have shown considerable shift, with the *MBR* being 73.9 in older movies, to almost achieving parity (54.5) in newer movies.

Associating beauty with fair skin: *RQ 1.3*

RQ 1.3*: Is beauty associated with fair skin in the movie dialogues describing women?*

Previous studies have found that on social media and dating sites, women are often judged by their appearance, whereas men are mostly judged by their behavior.[74–76] Skin color biases have been reported in the context of fairness beauty products in India,[14] Indian arranged marriages,[15] and surprisingly, political outcomes in India.[16]

We first present our cloze test results with the probe "*A beautiful woman should have* `[MASK]` *skin.*" in Table 5. We note that while $\texttt{BERT}_{base}$ model's top prediction is `soft`, all fine-tuned `BERT` models on the film corpora predict `fair` as the top choice. Figure 6 visualizes the nearest neighbors of `beautiful` in our aligned embedding spaces of Hollywood and Bollywood sub-corpora. As shown in Figure 6 (and Table 5), the age-old affinity toward lighter skin in Indian culture[77–79] is reflected through the consistent presence of `fair` among the nearest neighbors of all three Bollywood sub-corpora. Although our cloze tests indicate Hollywood also exhibits bias toward lighter skin color, our diachronic word embedding analysis reveals that possibly the bias is less pronounced than that in Bollywood.

Perception of dowry: *RQ 1.4*

As described in the gender attitudes and biases section, the dowry system involves a transaction of financial assets between

Table 6. Religious distribution of six major religions in India according to decennial census conducted in 1951, 1961, 1971, 1981, 1991, 2001, and 2011

Religion	1951 (%)	1961 (%)	1971 (%)	1981 (%)	1991 (%)	2001 (%)	2011 (%)
Hinduism	84.1	83.4	82.7	82.6	81.5	80.5	79.8
Islam	9.8	10.7	11.2	11.4	12.6	13.4	14.2
Christianity	2.3	2.4	2.6	2.4	2.3	2.3	2.3
Sikhism	1.9	1.8	1.9	2.0	1.9	1.9	1.7
Buddhism	0.7	0.7	0.7	0.7	0.8	0.8	0.7
Jainism	0.5	0.5	0.5	0.5	0.4	0.4	0.4

CellPress
OPEN ACCESS

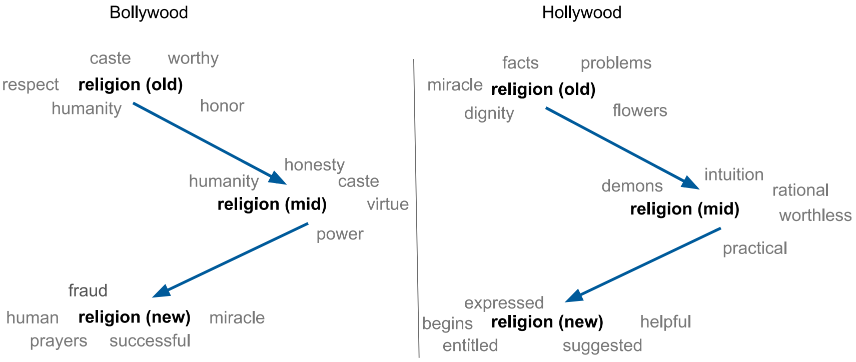

Figure 8. Nearest neighbors of `religion` over the years

Old, *mid*, and *new* denote the time periods 1950–69, 1970–99, and 2000–20, respectively.

the bride's family and the bridegroom's family, with the latter being the recipient of the financial assets. Understandably, this retrograde system can influence son preference, as a girl child might be looked upon as financial burden.[17] Despite legal prohibition since 1961,[19] this practice has continued in India with several studies linking it to other social crises such as female feticide,[20] domestic abuse and violence,[21,22] and dowry deaths.[23]

As shown in Figure 7, we observe that while nouns such as `money`, `debt`, `jewellery`, `fees`, and `loan` are the nearest neighbors in older films, indicating compliance to this practice, modern films exhibit non-compliance (e.g., `guts` and `refused`) and indicate some of the consequences of such non-compliance (e.g., `divorce` and `trouble`) in the form of nearest neighbors. Our findings align with a recent study based on a survey conducted among 4,603 women in Bihar (an Indian state in which the dowry has strong roots in tradition) that has reported positive changes in the society where the general attitude toward the dowry system has become negative.[24]

Religion

***RQ 2:** How are religions perceived in movies? Can we gain an insight into the religious representation of a country through a film corpus spanning 70 years?*

Perception of religion

Table 6 summarizes the religious distribution of six major religions in India according to the decennial censuses conducted since 1951. As indicated in Table 6, Hinduism and Islam are the two major religions in India, accounting for more than 90% of the country's population. Two major partitions in the last 70 years faced by the Indian subcontinent have resulted in considerable religious turmoil and riots between these two communities.[28]

The Central Board of Film Certification in India is a governing body that, along with giving each movie a certification, has the ability to remove offensive or controversial content, or in some extreme cases, it can completely ban films from being screened in theaters. With religion being a contentious topic in India, offensive terms surrounding it are also discouraged in films, and this has been constant throughout the years. To validate this hypothesis, we first look at the nearest neighbors of the word `religion` in the historical embeddings. Figure 8 indicates that `religion` is always accompanied with neutral or mild terms, and movie dialogues in Bollywood have stayed away from using extreme or hateful terms surrounding religion.

We next focus on the nearest neighbors of `Hindu` and `Muslim` in the historical embeddings and contrast our findings with prior research on aggregating social media perception of these two communities as presented in Palakodety et al.[31] As shown in Figures 9A and 9B, we find that while negative words like `ruthless`, `shameless`, and `traitor` creeping up in newer movies might indicate religious polarization, words like `terrorists` found in social media data from Palakodety et al.[31] are yet to surface among the nearest neighbors. Along with word embedding analysis, we analyze the `BERT` cloze tests for the probes (1) *Hindus are* `[MASK]` and (2) *Muslims are* `[MASK]`. For both probes, we do not notice completions such as `terrorists` or `fools` previously reported in Palakodety et al.[31] This suggests

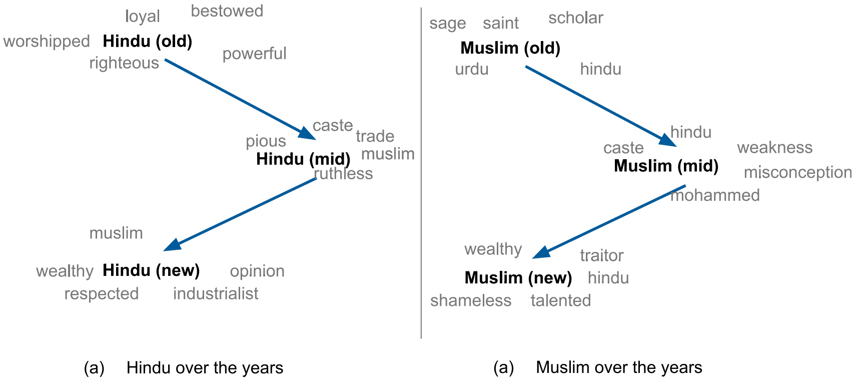

Figure 9. Nearest neighbors of `Hindu` and `Muslim` over the years

Old, *mid*, and *new* denote the time periods 1950–69, 1970–99, and 2000–20, respectively.

Table 7. Highly frequent surnames occurring in Bollywood movies (in decreasing order of frequency)

Most-frequent surnames
Singh, Krishna, Khan, Rai, Ali, Kapoor, Sharma, Mohan, Prasad, Khanna, Shah, Lal, Thakur, Dev, Shekhar, Chaudhary, Gandhi, Verma, Gupta, Prakash, Rana, Nath, Patel, Pandey, Roy, Pandit, Saxena, Mathur, Roshan, Bachchan, Pal, Mehta, Narayan, Das, Rode, Dayal, Mehra, Bhagat, Shastri, Chandra, Patil, Banerjee, Tilak, Rao, Tripathi, Yadav, Kumari, Suman, Mukherjee, Bhatia, Acharya, Chatterjee, Rehman, Iyer

that although recent social media analyses might indicate religious polarization, the film certification board has largely ensured movie content does not reflect such an extreme divide.

Religious representation

While the religious composition in India largely remained stable over the years, Table 6 indicates a slow decline in the population share of Hinduism and an increase of population share of Islam. In fact, the shifting nature of the demographic balance between the two major religions in India and its possible interpretations have a long history of use in political debates.[26] We conduct a comprehensive analysis of the evolving nature of religious representation in Bollywood content through surname usage in our data set (Table 7 lists a set of highly frequent surnames occurring in Bollywood movies; details are presented in experimental procedures). Figure 10 contrasts the religion distribution obtained in movies with ground truth census data.

We note the following: (1) the distribution is more or less consistent with the census numbers; (2) representation for other religions has increased in recent years; and (3) the representation of Muslims is slightly less than the community's population share.

Table 8 indicates the surnames of the doctors occurring in Bollywood movies. To retrieve these surnames from the subtitles, we employ a template-based approach, searching for keywords like `"Dr."` and `"doctor"` from our corpus. While a broader religious representation is observed in our overall results, we find that the representation for the medical profession is quite skewed, with a large number of surnames being Brahmins (the uppermost caste in the Hindu caste system[80] in India).

Attitudes and biases toward geographic regions

RQ 3: *How has geographic representation evolved over time in Bollywood content? Which geographic areas have been consistently under-represented in the Mumbai film industry?*

As already discussed (in the attitudes and biases toward geographic regions section), similar to religion, regional politics has been a recurrent theme in Indian political discourse.[5] From the beginning, Bollywood has had its roots in Mumbai, and Delhi is India's capital. Hence, it is not surprising that these cities are mentioned heavily across all time periods (see Table 9). Figures 11A and 11B compare the geographic representations in the most recent 20 years with the rest of our corpus spanning 1950–99. We observe that initially based out of major hotspots of Delhi, Goa, and cities like Mumbai, recent Bollywood content

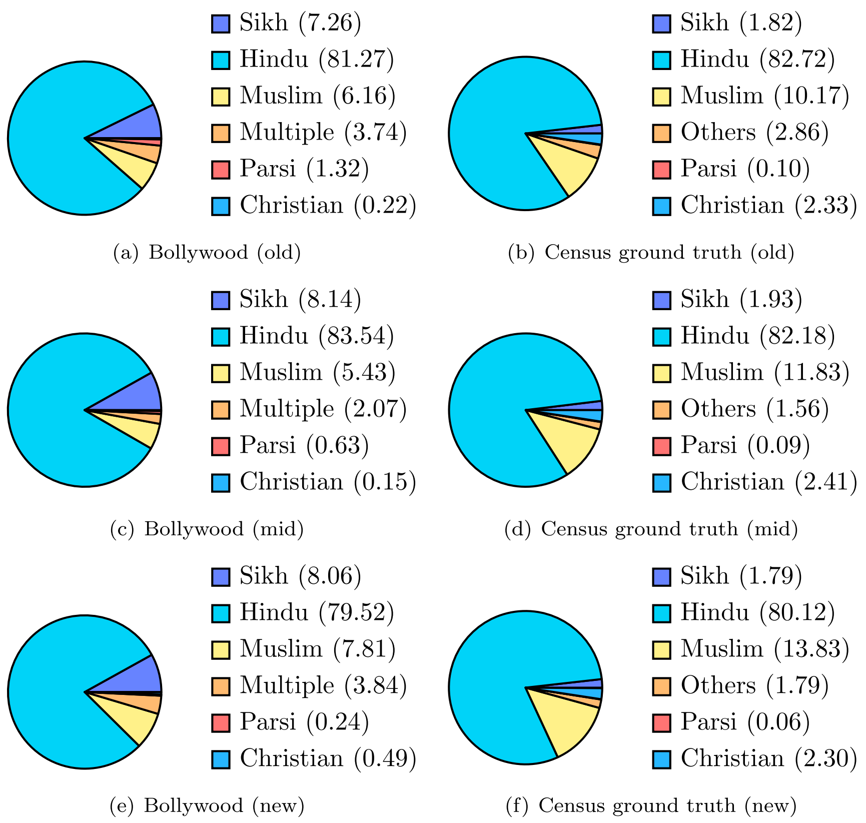

Figure 10. Religious representation in Bollywood movies (left) contrasted with ground truth census data (right)
Old, *mid*, and *new* denote the time periods 1950–69, 1970–99, and 2000–20, respectively.

Table 8. Surnames of doctors in Bollywood movies

Surnames of doctors
Kapur, Chopra, Khurana, Tripathi, Kapoor, Ansari, Awasthi, Kothari, Mathur, Puri, Nayak, Bhalerao, Sawant, Tandon, Swamy, Banerjee, Verma, Rana, Ruby, Singh, Shrivastav, Khanna, Bhandari, Tiwari, Saxena, Shinde, Mehta, Goenka, Kumar, Goswami

is geographically more diverse and inclusive. However, a key point we highlight in Figure 11C is that, in line with prior research on under-representation of northeastern states in news content,[30] there is severe under-representation of these northeastern states in Bollywood content. In fact, there have been zero mentions of the states of Arunachal Pradesh, Meghalaya, and Mizoram in over 700 movies across 70 years

Economic signals and national priorities

RQ 4: *Can we extract economic signals through popular film dialogues? Can we track evolving national priorities from popular entertainment?*

Economic signals

By looking at a popular entertainment corpus of a developing nation, we are able to showcase the evolution of gender bias, evolving attitudes toward social evils, and geographic and religious representations. Can we detect economic signals as well from the Bollywood dialogues? 100 rupees in 1958 is equivalent to 8,117.22 rupees in 2020 (https://www.inflationtool.com/indian-rupee). We seek to understand whether language models can capture these noisy signals. `GPT-2`,[59] a popular language model with more than 100 million parameters, has achieved state-of-the-art results for text completion, zero shot transfer learning, etc. `GPT-2` has been widely used for generating free form text to create artificial newsletters, poems, etc. (https://www.gwern.net/GPT-2). We noticed that the most common dialogues expressing monetary figures or large amounts of money were generally associated with ransom. For example, a sample dialogue is "We have kidnapped your kid; the ransom amount is 2 million rupees."

To analyze the historical trends, we fine-tune `GPT-2` on three Bollywood sub-corpora, each belonging to films from different time periods, for the end goal of free form text completion. On these fine-tuned models, we input the sentence *"The ransom amount is"* and analyze the generated text by the model. Table 10 showcases the average amount across 100 generated samples from the fine-tuned models. We note that while our predicted values overestimate the inflation rate, the ransom amounts capture the general increasing pattern and have increased significantly over time.

Evolving national priorities

Similar to `BERT`'s cloze test applications to uncover occupational stereotypes and bias toward fair skin color, following Palakodety et al. and KhudaBukhshet al.,[31,32] we employ `BERT` to analyze evolving national priorities using the following two cloze tests:

1. *The biggest problem in India is* `[MASK]` (for Bollywood).
2. *The biggest problem in America is* `[MASK]` (for Hollywood).

We observe that the dynamic political conditions are reflected in the completion results in Tables 11 and 12 (e.g., `Kashmir`, `Pakistan`, and `Russia`).[65,66,81] We also note that the list of ongoing problems in the United States contains the major issue in the 2020 election: racism.

DISCUSSION

In this paper, we analyzed how social biases and subtle gender biases get reflected on diachronic corpora of popular entertainment. Our research indicates that our NLP methods are capable of uncovering important social signals. Some of the findings in our papers are anecdotally *known* or *expected*. For instance, it is not difficult to envision that award-winning, critically acclaimed world movies may exhibit more progressive attitudes toward gender equality than Bollywood potboilers. And, it is not surprising that male-dominated action movies would exhibit larger gender bias than romantic films. Many of our research questions have been explored in prior social science literature. However, such efforts are typically limited to a handful of films (see, e.g., Dimitrova, Rao, and Lundberg[4,6,82]). However, what we offer here is a comprehensive, quantitative, and large-scale analysis of these research questions using a suite of cutting-edge NLP techniques in a synergistic way. Our methods thus allow us to measure more objectively and quantitatively. They also allow precise tracking of change in these biases over time.

Large-scale analyses of texts are intractable without automated methods. We present statistical, automated analysis of movies at scale and across time that gives us a finer probe for understanding the cultural themes implicit in these films. In our

Table 9. City mentions in movies from our Bollywood corpus. *Old*, *mid*, and *new* denote the time periods 1950–1969, 1970–1999, and 2000–2020, respectively

Old	*Mid*	*New*
Bombay/Mumbai (51)	Bombay/Mumbai (68)	Bombay/Mumbai (83)
Delhi (27)	Delhi (45)	Delhi (52)
Kolkata/Calcutta (23)	Kolkata/Calcutta (18)	Amritsar (9)
Lucknow (14)	Lucknow (12)	Bangalore/Bengaluru (9)
Madras/Chennai (10)	Simla/Shimla (12)	Kolkata/Calcutta (9)
Agra (6)	Madras/Chennai (10)	Pune (8)
Srinagar (6)	Pune (8)	Lucknow (7)
Simla/Shimla (6)	Bangalore/Bengaluru (7)	Hyderabad (6)
Mathura (5)	Nagpur (6)	Madras/Chennai (6)

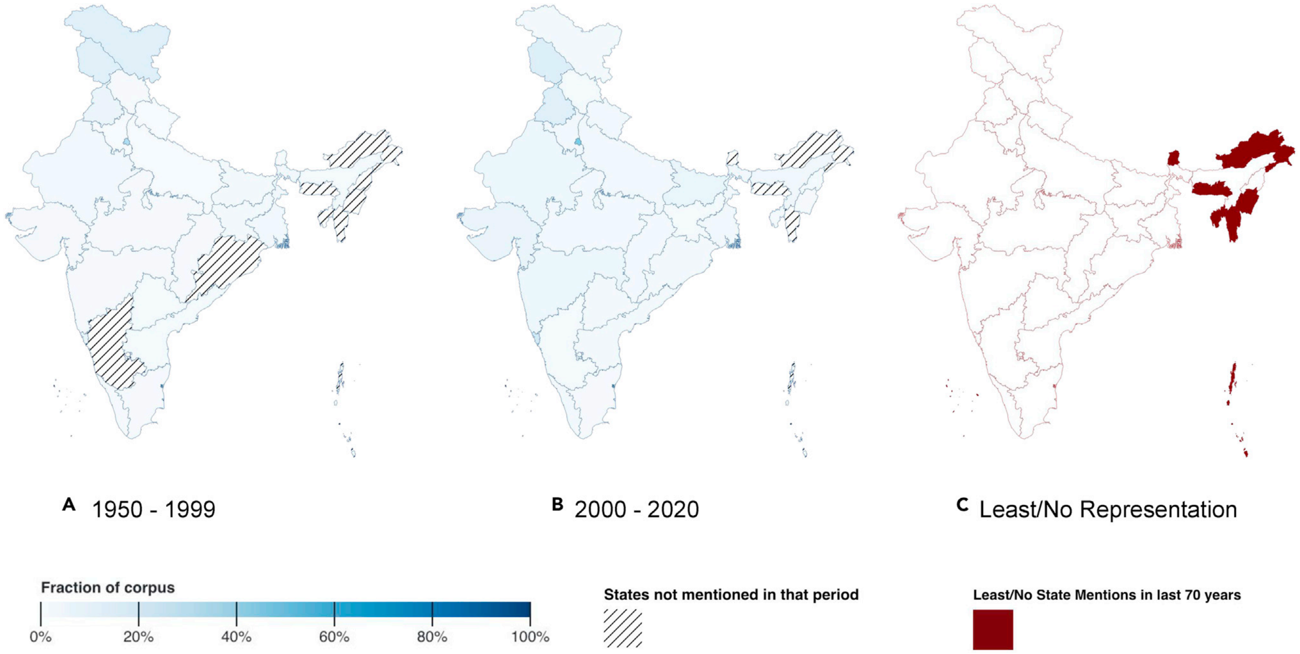

Figure 11. Geographic representation in Bollywood movies
(A) Geographical representation in films during the period 1950–1999.
(B) Geographical representation in films post 2000.
(C) States with least or no representation (less than 0.2% movies in the entire corpus) in our corpus in the last 70 years. The base maps used for this plot are sourced from the Government of India. The authors are aware that these maps include disputed territories. These maps do not constitute judgments on existing disputes.

study, we restrict ourselves to 100 movies per decade. However, the methods presented in this paper can easily scale up to 1,000 or more movies as long as we have English subtitles for them. Also, the same NLP tools might be used to rapidly analyze hundreds or thousands of books, magazine articles, radio transcripts, or social media posts.

Not all the uncovered biases are known. For instance, our analyses indicate that babies born inside popular Bollywood films exhibit a skewed gender distribution. This gender disparity has evolved (and improved) over time. Without a large-scale analysis, these types of insights are hard to obtain.

When contrasted with real-world data, we observe that our findings in movies somewhat align to real-world data. However, sometimes movies show better *numbers* than present in the society, and sometimes the numbers are worse. For example, in our analyses, we observe that the representation of Muslims in Bollywood movies is slightly less than the community's population share. In the case of son preference, our results indicate an improving gender ratio (MBR) over time. However, in reality, the 2011 census recorded an all-time-low CSR. Similarly, our economic signal overestimates the actual inflation rate while retaining the general increasing pattern. Our geographic representation results also corroborate the historical under-representation of northeastern states in other media.[30]

With the advent of the multiplex era in Indian cinema, different movies can offer different prices. Hence, if a progressive idea has enough takers, movies exhibiting that progressive idea can generate substantial revenue. When certain numbers are *better* in movies than in the society, we hypothesize that this is a signal that society could be receptive to the idea, and that we might see real numbers in society improve over the coming years. (In fact, a recent study has revealed that some of the most imbalanced districts in India are on their way to recording better CSR numbers.[83]) In contrast, when numbers in movies are worse than society, this could be a signal that the society is less receptive to the progressive idea.

Our results demonstrate that societal changes do get reflected in popular content. But does popular entertainment also influence the society in turn? A recent movie on acid attack, Chhapak, was inspired from a true story of an acid attack survivor who set up an NGO and was a recipient of the International Women of Courage[84] award. Her biopic and her initiative of *Stop Acid Sale* when released, triggered regulatory legislation that made it difficult to buy certain types of acids without legal authorization.

Table 10. Average amount for text completion results on the input sentence "*The ransom amount is*" using fine-tuned `GPT-2` models

	Old	*Mid*	*New*
Predicted ransom amount	594,805 ± 43,159	10,959,940 ± 123,217.34	29,688,280 ± 119,544.28
Inflation-adjusted amount	–	2,194,830	21,000,280

The inflation-adjusted values for 594,805 INR in 1960 are presented in the bottom row.

CellPress
OPEN ACCESS

Table 11. Cloze test results for *The biggest problem in India is* [MASK]

$BERT_{base}$	$BERT_{\mathcal{D}^{old}_{bolly}}$	$BERT_{\mathcal{D}^{old}_{bolly}}$
corruption (0.034), poverty (0.020), malaria (0.019), pollution (0.012), hunger (0.012), terrorism (0.009), unemployment (0.008), drought (0.008), famine (0.007), war (0.003), tourism (0.001)	poverty (0.078), love (0.072), war (0.067), hunger (0.049), unemployment (0.043), India (0.042), famine (0.029), money (0.023), marriage (0.012), education (0.011), Kashmir (0.009)	poverty (0.074), Pakistan (0.072), Kashmir (0.053), terrorism (0.051), corruption (0.037), India (0.031), drugs (0.021), dowry (0.016), unemployment (0.014), hunger (0.009), rape (0.006)

Devising NLP methods to identify how popular entertainment influences society will be a worthy future research challenge.

Limitations

Our study has several limitations. For some of our experiments, we build upon pre-trained language models trained on vast amount of texts. While our experiments on diachronic word embeddings and WEAT train the word embeddings from scratch, our experiments using BERT and GPT-2 are trained on top of pre-trained models. Recent works have indicated that these models have a wide range of biases that reflect the texts on which they were originally trained, and which may percolate to downstream tasks.[85]

Our study focuses on linguistic signals obtained from English subtitles of popular films. However, films have a strong visual component, and linguistic signals may not be able to capture biases present in the visual medium. For example, Dhoom 2, a commercially successful movie released in 2006, belongs to our data set. In one of the scenes in this film, Ali, one of the main characters in the film, fantasizes a future family with Monali, another character in the film. In this sequence with screen time of less than 10 s and without any dialogue, Ali's dream family indicates that the two of them (Ali and Monali) have four children, where all of them are sons. Our analysis of son preference relying solely on movie dialogues will be unable to capture this subtle visual signal hinting deep-seated son preference. Our study will substantially benefit from a multi-modal analysis considering both linguistic and visual signals.

Finally, Bollywood represents a fraction of Indian cinema. There are many regional language movie industries (e.g., Kannada, Telugu, Tamil, Malayalam, Bengali, and Marathi) that heavily contribute to Indian cinema. According to the Central Board of Film Certification report (http://www.filmfed.org/downloads/Language-wise-Region-2018-19-26062019.pdf), out of the 1,966 movies certified by the board in 2019, only 495 movies (24.92%) came from the Mumbai film industry. Hence, extending our study to regional movies will strengthen our analyses further. Also, the location of Mumbai and linguistic barrier may influence some of our analyses. For example, in our geographic representation analysis, we found minuscule representation of several northeastern states. While these states have documented under-representation in other media such as the print news medium, it is possible that linguistic barrier and geographic distance were potential factors.

EXPERIMENTAL PROCEDURES

Resource availability

Lead contact

Requests for data and requests for additional information should be directed to the lead contact, Ashiqur R. KhudaBukhsh (axkvse@rit.edu).

Materials availability

This study did not generate physical materials.

Data and code availability

Data and code are available at https://github.com/kunalkhadilkar/CellPatternsBollywood.

Data set

We construct the following two data sets of movie subtitles.

1. **Bollywood movies,** D_{bolly}**:** We consider 100 top-grossing movies for each decade spanning 1950–2020 (7 decades, and overall, 700 movies). We retrieve English subtitles[86] for each of these 700 movies.
2. **Hollywood movies,** D_{holly}**:** Similar to Bollywood movies, we consider 100 top-grossing movies from each of the seven decades (700 total films). Overall, $\mathcal{D}_{bolly}$ and $\mathcal{D}_{holly}$ consist of 1.1M dialogues (6.2M tokens) and 1M dialogues (5.4M tokens), respectively.

In several experiments, we divide our corpus into three temporal buckets, presented in Tables 13 and 14. Our choice of separation points in the timeline is guided by the global emergence of counter-culture in the late 60s and early 70s[87] and the rapid rise of multiplex culture in Indian cinema.[88] We understand that several other reasonable binning choices exist. In the supplemental information (Table S3), we have shown experimental results that indicate that our qualitative claims remain unchanged with an alternative binning.

We further collect 150 movies that have been nominated for the Best International Feature Film award 1970 onward at the Oscars (https://www.oscars.org) for a subset of our analyses.

Cloze test and free form completions

In our experiments with language models, we use both BERT and GPT-2 because of their different capabilities. For instance, BERT is particularly well-suited for cloze tests, and there exists substantial literature where BERT has

Table 12. Cloze test results for *The biggest problem in America is* [MASK]

$BERT_{base}$	$BERT_{\mathcal{D}^{old}_{bolly}}$	$BERT_{\mathcal{D}^{old}_{bolly}}$
poverty (0.076), corruption (0.072), unemployment (0.061), crime (0.045), terrorism (0.042), racism (0.027), pollution (0.021), hunger (0.016), war (0.012), cancer (0.009), inequality (0.003)	war (0.092), poverty (0.083), money (0.062), unemployment (0.053), slavery (0.051), immigration (0.045), alcoholism (0.041), education (0.032), imperialism (0.023), Russia (0.023), hunger (0.019)	poverty (0.088), slavery (0.082), immigration (0.078), unemployment (0.073), money (0.071), war (0.065), racism (0.053), hunger (0.024), communism (0.016), America (0.011), education (0.006)

Table 13. Data set splits for Bollywood

Corpus	Industry	Time period
$\mathcal{D}^{old}_{bolly}$	Bollywood	1950–69
$\mathcal{D}^{mid}_{bolly}$	Bollywood	1970–99
$\mathcal{D}^{new}_{bolly}$	Bollywood	2000–20

Table 14. Data set splits for Hollywood

Corpus	Industry	Time period
$\mathcal{D}^{old}_{holly}$	Hollywood	1950–69
$\mathcal{D}^{mid}_{holly}$	Hollywood	1970–99
$\mathcal{D}^{new}_{holly}$	Hollywood	2000–20

been deployed for this task.[31,56,68,69] On the other hand, for our economic signal mining task, the ransom money can be specified as a free form text (e.g., 0.5 million dollars or 500 grands). `GPT-2` is particularly well-suited for free form text completions. Moreover, the outputs of `GPT-2` are non-deterministic, which allows us to conduct multiple runs of the same experiment and compute confidence intervals.

We follow the standard preprocessing steps recommended to fine-tune `BERT` language model. For our task, we use the `bert-base-uncased` pre-trained English model, with the following parameter details: 12 transformer layers, hidden state length of 768, 12 attention heads, and 110M overall parameters (denoted as $\texttt{BERT}_{base}$). The pre-trained model is fine-tuned on the target corpus using the training parameters showcased below.

- batch size: 16
- maximum sequence length: 128
- maximum predictions per sequence: 20
- fine-tuning steps: 10,000
- warmup steps: 10
- learning rate: 2e–5

For fine-tuning the language model for free form text completion tasks, we use the smallest `GPT-2` model with 124M parameters, trained for 10,000 steps.

WEAT

We follow the experimental protocols identical to those specified in Van Miltenburg,[34] the paper that introduced this technique. Following Van Miltenburg,[34] we train the sub-corpora using GloVe embeddings.[89] We consider two equal sized sets of occupations, S_1 and S_2, and two sets of attribute words, A_1 and A_2.

The similarity of two words, say x and y, is given by calculating the cosine similarity of the corresponding word embeddings, $cos(w_x, w_y)$. As given in Van Miltenburg,[34] the differential association of a word c with word sets A_1 and A_2 is given by the following:

$$g(c, A_1, A_2, w) = \underset{a}{\overset{mean}{}} \in A_1 cos(w_c, w_a) - \underset{b}{\overset{mean}{}} \in A_2 cos(w_c, w_b) \quad \text{(Equation 1)}$$

Next, the `WEAT` score is calculated:

$$B_{weat}(w) = \frac{\underset{s}{\overset{mean}{}} \in S_1 g(s, A_1, A_2, w) - \underset{t}{\overset{mean}{}} \in S_2 g(t, A_1, A_2, w)}{\underset{s}{\overset{std-dev}{}} \in S_1 S_2 g(s, A_1, A_2, w)} \quad \text{(Equation 2)}$$

The occupation sets, S_1 and S_2, are taken from Bolukbasi et al.:[41]

S_1 = {`maestro`, `skipper`, `protege`, `philosopher`, `captain`, `architect`, `financier`, `warrior`, `broadcaster`, `magician`, `pilot`, `boss`}.

S_2 = {`homemaker`, `nurse`, `receptionist`, `librarian`, `socialite`, `hairdresser`, `nanny`, `bookkeeper`, `stylist`, `housekeeper`, `designer`, `counselor`}.

The attribute word sets A_1 and A_2 are A_1 = {`he`, `man`, `male`} andA_2 = {`she`, `woman`, `female`}.

In order to investigate if the `WEAT` results are mere artifacts of the embedding model or not, we conduct the following experiment. We modify the corpora by randomly flipping the gendered pronouns in the corpus and then train the embedding models and recompute the `WEAT` scores. We find that the `WEAT` score is close to zero (0.0158 ± 0.039). Thus the bias present in the model is minimal, and our obtained results are reliable.

Aligning diachronic word embeddings

We use a robust multilingual approach to align diachronic word embeddings using orthogonal Procrustes (estimating an orthogonal matrix that maps one set of points to another) as described in Hamilton et al.[58] We follow the same method to align different sub-corpora for Bollywood and Hollywood. For each sub-corpora listed in Tables 13 and 14, we train word2vec[90] with SGNS (skip-gram with negative sampling) to obtain word embeddings. Let $\mathcal{W}^{(t)} \in \mathbb{R}^{(d) \times \mathcal{V}}$ be the matrix of word embeddings learnt for period t for vocabulary $\mathcal{V}$. Following Mikolov,[90] we align the word embeddings using the top 10,000 common tokens present across time periods t and $t+1$ by optimizing:

$$R^t = argmin_{Q^T Q = I} \parallel QW^t - W^{(t+1)} \parallel_F \quad \text{(Equation 3)}$$

where $R^t \in \mathbb{R}^{d \times d}$.

Note that, our choices of word embeddings for our experiments to compute `WEAT` score and to align diachronic word embeddings differ because in all our experiments, for each of the techniques, we have followed experimental protocols identical to those specified in the papers that introduced these techniques.

Son preference

We retrieve the dialogues talking about childbirth using a template-based approach, by searching for the following keywords and phrases: `birth`, `baby`, `pregnant`, `pregnancy`, `congratulations`, `"It's a boy,''` and `"It's a girl.''` We annotated the retrieved dialogues related to childbirth and performed a temporal analysis.

Religious representation

437 surnames appearing in the movies from our corpus (e.g., Mrs. Kapoor, Mr. Khan, etc.) are annotated manually by two annotators, with each surname given one label from the list of labels: Hindu, Muslim, Sikh, Christian, Parsi, or multiple. Since almost all Jain surnames are also highly prominent Hindu surnames (e.g., Mehta, Chopra), and unlike several major religions, Buddhism does not have easy-to-distinguish last names, we exclude Jainism and Buddhism in our analysis. The annotators achieved a Cohen κ score of 0.8879, indicating high inter-rater agreement. The discrepancies were resolved by the annotators through a follow-up adjudication process and by consulting relevant literature (e.g., tracking biographies of prominent personalities having a specific last name). A random sample of prominent Indian surnames annotated with religions is presented in the supplemental information (Table S2).

SUPPLEMENTAL INFORMATION

Supplemental information can be found online at https://doi.org/10.1016/j.patter.2021.100409.

ACKNOWLEDGMENTS

We are thankful to Parichay Patra for his insightful feedback. We express our gratitude to the anonymous reviewers for their thoughtful, valuable, and constructive comments.

AUTHOR CONTRIBUTIONS

A.K. conceived the idea, approach, and research questions; K.K. collected the data set and performed experiments; A.K and T.M. designed the experiments; A.K., K.K., and T.M. wrote the manuscript.

DECLARATION OF INTERESTS

The authors declare no competing interests.

CellPress
OPEN ACCESS

Received: May 13, 2021
Revised: October 14, 2021
Accepted: November 16, 2021
Published: December 9, 2021; Corrected online: January 18, 2022

REFERENCES

1. Khadilkar, K., and KhudaBukhsh, A.R. (2021). An unfair affinity toward fairness: characterizing 70 years of social biases in B^{H}ollywood (student abstract). In Thirty-Fifth AAAI Conference on Artificial Intelligence, AAAI 2021 (AAAI Press), pp. 15813–15814. https://ojs.aaai.org/index.php/AAAI/article/view/17903.
2. Jaffrelot, C. (2010). Religion, Caste, and Politics in India (Primus Books).
3. Zargar, H. (2020). How Bollywood furthers India's Hindu nationalism. https://www.newframe.com/how-bollywood-furthers-indias-nationalism/.
4. Dimitrova, D. (2010). Religion and gender in bollywood film. In Religion in Literature and Film in South Asia (Springer), pp. 69–81.
5. Ramaswamy, S. (1997). Passions of the Tongue: Language Devotion in Tamil India, 1891–1970, *Vol. 29* (Univ of California Press).
6. Rao, L. (1989). Woman in Indian films: a paradigm of continuity and change. Media, Cult. Soc. *11*, 443–458.
7. Twenge, J.M., Campbell, W.K., and Gentile, B. (2012). Male and female pronoun use in US books reflects women's status, 1900–2008. Sex Roles *67*, 488–493.
8. Lahoti, R., and Swaminathan, H. (2016). Economic development and women's labor force participation in India. Feminist Econ. *22*, 168–195.
9. Garg, N., Schiebinger, L., Jurafsky, D., and Zou, J. (2018). Word embeddings quantify 100 years of gender and ethnic stereotypes. Proc. Natl. Acad. Sci. U S A *115*, E3635–E3644.
10. R. Pande, A. Malhotra, S. Mathur, M. Mehta, A. Malhotra, M.A. Lycette, S.D. Kambou, V. Magar, J. Gay, H. Lary, et al., Son Preference and Daughter Neglect in India.International Center for Research on Women
11. Bhat, P.N.M., and Zavier, A.J.F. (2003). Fertility decline and gender bias in Northern India. Demography *40*, 637–657. http://www.jstor.org/stable/1515201.
12. R. Echávarri, Gender Bias in Sex Ratio at Birth: The Case of India.Universidad Pública de Navarra
13. Mukherjee, S.S. (2013). Women's empowerment and gender bias in the birth and survival of girls in urban India. Feminist Econ. *19*, 1–28. https://www.tandfonline.com/doi/abs/10.1080/13545701.2012.752312.
14. N. Shevde, All's fair in love and cream: a cultural case study of fair & lovely in India, Advertising Soc. Rev. 9 10.1353/asr.0.0003
15. Nagar, I. (2018). The unfair selection: a study on skin-color bias in arranged Indian marriages. Sage Open *8*. https://doi.org/10.1177/2158244018773149.
16. Ahuja, A., Ostermann, S.L., and Mehta, A. (2016). Is only fair lovely in indian politics? Consequences of skin color in a survey experiment in Delhi. J. Race, Ethn. Polit. *1*, 227–252.
17. Diamond-Smith, N., Luke, N., and McGarvey, S. (2008). 'Too many girls, too much dowry': son preference and daughter aversion in rural Tamil Nadu, India. Cult. Health Sex. *10*, 697–708.
18. Dalmia, S., and Lawrence, P.G. (2005). The institution of dowry in India: why it continues to prevail. J. Develop. Areas, 71–93.
19. Rao, R.J. (1973). Dowry system in India — a socio-legal approach to the problem. J. Indian L. Inst. *15*, 617–625.
20. Ghansham, D.M. (2002). Female foeticide and the dowry system in India. In Townsville International Women's Conference (James Cook Univ.).
21. Banerjee, P.R. (2014). Dowry in 21st-century India: the sociocultural face of exploitation. Trauma, Violence, & Abuse *15*, 34–40.
22. Rastogi, M., and Therly, P. (2006). Dowry and its link to violence against women in India: feminist psychological perspectives. Trauma, Violence, & Abuse *7*, 66–77.
23. Ahmad, N. (2008). Dowry deaths (bride burning) in India and abetment of suicide: a socio-legal appraisal. JE Asia Int'l L. *1*, 275.
24. Srinivasan, P., and Lee, G.R. (2004). The dowry system in Northern India: women's attitudes and social change. J. Marriage Fam. *66*, 1108–1117.
25. Rao, V. (1993). The rising price of husbands: a hedonic analysis of dowry increases in rural India. J. Polit. Econ. *101*, 666–677.
26. Pethe, V.P. (1973). Hindus, muslims and the demographic balance in India. Econ. Polit. Weekly *8*, 75–78.
27. Talbot, I., and Singh, G. (2009). The Partition of India (Cambridge University Press).
28. Iyer, S., and Shrivastava, A. (2018). Religious riots and electoral politics in India. J. Develop. Econ. *131*, 104–122.
29. Majumdar, B. (2006). When north–south fight, the nation is out of sight: the politics of olympic sport in postcolonial India. Int. J. Hist. Sport *23*, 1217–1231.
30. Chongloi, H. (2017). Portrayal of northeast India in mainstream media: a case of underrepresentation and misinterpretation. Int. J. Res. Social Sci. *7*, 613–623.
31. Palakodety, S., KhudaBukhsh, A.R., and Carbonell, J.G. (2020). Mining insights from large-scale corpora using fine-tuned language models. In Proceedings of the Twenty-Fourth European Conference on Artificial Intelligence (ECAI-20), pp. 1890–1897.
32. R. KhudaBukhsh, R. Sarkar, M.S. Kamlet, T.M. Mitchell, Fringe news networks: dynamics of US news viewership following the 2020 presidential election, arXiv:2101.10112.
33. Van Miltenburg, E. (2016). Stereotyping and bias in the flickr30k dataset. arXiv, 1605.06083.
34. Caliskan, R., Bryson, J.J., and Narayanan, A. (2017). Semantics derived automatically from language corpora contain human-like biases. Science *356*, 183–186.
35. Lewis, M., and Lupyan, G. (2020). Gender stereotypes are reflected in the distributional structure of 25 languages. Nat. Hum. Behav. *4*, 1–8.
36. Prates, M.O., Avelar, P.H., and Lamb, L.C. (2019). Assessing gender bias in machine translation: a case study with google translate. Neural Comput. Appl. *32*, 1–19.
37. Brown, T.B., Mann, B., Ryder, N., Subbiah, M., Kaplan, J., Dhariwal, P., Neelakantan, A., Shyam, P., Sastry, G., Askell, A., et al. (2020). language models are few-shot learners. In Advances in Neural Information Processing Systems 33: Annual Conference on Neural Information Processing Systems 2020, NeurIPS 2020, December 6–12, virtual https://proceedings.neurips.cc/paper/2020/hash/1457c0d6bfcb4967418bfb8ac142f64a-Abstract.html.
38. Lucy, L., Demszky, D., Bromley, P., and Jurafsky, D. (2020). Content analysis of textbooks via natural language processing: findings on gender, race, and ethnicity in Texas U.S. history textbooks. AERA Open *6*. https://doi.org/10.1177/2332858420940312.
39. Gala, D., Khursheed, M.O., Lerner, H., O'Connor, B., and Iyyer, M. (2020). Analyzing gender bias within narrative tropes. arXiv, 2011.00092.
40. Kumar, V., Bhotia, T.S., Kumar, V., and Chakraborty, T. (2020). Nurse is closer to woman than surgeon? Mitigating gender-biased proximities in word embeddings. Trans. Assoc. Comput. Linguistics *8*, 486–503. https://doi.org/10.1162/tacl_a_00327.
41. Bolukbasi, T., Chang, K.-W., Zou, J.Y., Saligrama, V., and Kalai, A.T. (2016). Man is to computer programmer as woman is to homemaker? debiasing word embeddings. Adv. Neural Inf. Process. Syst. *29*, 4349–4357.
42. Manzini, T., Yao Chong, L., Black, A.W., and Tsvetkov, Y. (2019). Black Is to Criminal as Caucasian Is to Police: Detecting and Removing Multiclass Bias in Word Embeddings (NAACL-HLT), pp. 615–621.
43. Garrido-Muñoz, I., Montejo-Ráez, A., Martínez-Santiago, F., and Ureña-López, L.A. (2021). A survey on bias in deep NLP. Appl. Sci. *11*, 3184.
44. Chatterjee, S. (2016). 'English Vinglish' and Bollywood: what is 'new' about the 'new woman'? Gend. Place Cult. *23*, 1179–1192.

45. Khan, S., and Taylor, L. (2018). Gender policing in mainstream Hindi cinema: a decade of central female characters in top-grossing bollywood movies. Int. J. Commun. *12*, 22.
46. Madaan, N., Mehta, S., Agrawaal, T.S., Malhotra, V., Aggarwal, A., Gupta, Y., and Saxena, M. (2018). Analyze, detect and remove gender stereotyping from bollywood movies. In Conference on Fairness, Accountability and Transparency, FAT 2018, 23–24 February 2018, New York, NY, USA, Vol. 81 of Proceedings of Machine Learning Research, S.A. Friedler and C. Wilson, eds. (PMLR), pp. 92–105. http://proceedings.mlr.press/v81/madaan18a.html.
47. S. Sheth, G. Jones, M. Spencer, Bollywood, Skin Color and Sexism: The Role of the Film Industry in Emboldening and Contesting Stereotypes in India after Independence.Harvard Business School
48. Mishra, N. (2015). India and colorism: the finer nuances. Wash. U. Glob. Stud. L. Rev. *14*, 725.
49. Breakthrough (2019). 8 examples of nerve-wracking misogynist dialogues in bollywood. https://inbreakthrough.org/misogynist-dialogues-bollywood/.
50. Thesecondangle (2020). Top 10 sexist dialogues in bollywood. https://thesecondangle.com/sexist-dialogues-bollywood/.
51. Popxo (2019). Stupid and sexist: 20 WTF dialogues that prove bollywood is unfair towards women. https://www.popxo.com/2019/07/20-bollywood-dialogues-that-are-worst-examples-of-sexism/.
52. Warriner, B., Kuperman, V., and Brysbaert, M. (2013). Norms of valence, arousal, and dominance for 13,915 English lemmas. Behav. Res. Methods *45*, 1191–1207.
53. Sendén, M.G., Sikström, S., and Lindholm, T. (2015). "She" and "He" in news media messages: pronoun use reflects gender biases in semantic contexts. Sex Roles *72*, 40–49.
54. Taylor, W.L. (1953). "Cloze procedure": a new tool for measuring readability. Journalism Q. *30*, 415–433.
55. Smith, N., and Levy, R. (2011). Cloze but no cigar: the complex relationship between cloze, corpus, and subjective probabilities in language processing. Proceedings of the Annual Meeting of the Cognitive Science Society, *33*.
56. Petroni, F., Rocktäschel, T., Riedel, S., Lewis, P., Bakhtin, A., Wu, Y., and Miller, A. (2019). Language models as knowledge bases? In Proceedings of the 2019 Conference on Empirical Methods in Natural Language Processing and the 9th International Joint Conference on Natural Language Processing (EMNLP-IJCNLP) (Association for Computational Linguistics), pp. 2463–2473. https://doi.org/10.18653/v1/D19-1250. https://www.aclweb.org/anthology/D19-1250.
57. Devlin, J., Chang, M.-W., Lee, K., and Toutanova, K. (2019). BERT: Pre-training of Deep Bidirectional Transformers for Language Understanding (NAACL-HLT), pp. 4171–4186.
58. Hamilton, W.L., Leskovec, J., and Jurafsky, D. (2016). Diachronic word embeddings reveal statistical laws of semantic change. In Proceedings of the 54th Annual Meeting of the Association for Computational Linguistics (Volume 1: Long Papers) (Association for Computational Linguistics), pp. 1489–1501. https://doi.org/10.18653/v1/P16-1141. https://www.aclweb.org/anthology/P16-1141.
59. Radford, A., Wu, J., Child, R., Luan, D., Amodei, D., and Sutskever, I.. Language models are unsupervised multitask learners. https://d4mucfpksywv.cloudfront.net/better-language-models/language-models.pdf.
60. Bhargava, R. (2007). On the persistent political under-representation of Muslims in India. L. Ethics Hum. Rights *1*, 76–133.
61. Greenwald, G., and Banaji, M.R. (1995). Implicit social cognition: attitudes, self-esteem, and stereotypes. Psychol. Rev. *102*, 4.
62. Xie, J.Y., Ferreira Pinto Junior, R., Hirst, G., and Xu, Y. (2019). Text-based inference of moral sentiment change. In Proceedings of the 2019 Conference on Empirical Methods in Natural Language Processing and the 9th International Joint Conference on Natural Language Processing (EMNLP-IJCNLP) (Association for Computational Linguistics), pp. 4654–4663. https://doi.org/10.18653/v1/D19-1472. https://www.aclweb.org/anthology/D19-1472.
63. Hardgrave, R.L., Jr. (1985). India in 1984: confrontation, assassination, and succession. Asian Surv. *25*, 131–144.
64. Kaarthikenyan, D.R., and Raju, R. (2008). Rajiv Gandhi Assassination (Sterling Publishers Pvt. Ltd.).
65. Schofield, V. (2010). Kashmir in Conflict: India, Pakistan and the Unending War (Bloomsbury Publishing).
66. Bose, S. (2009). Kashmir: Roots of Conflict, Paths to Peace (Harvard University Press).
67. Pedersen, J.D. (2000). Explaining economic liberalization in India: state and society perspectives. World Develop. *28*, 265–282.
68. N. Kassner, P. Dufter, H. Schütze, Multilingual LAMA: investigating knowledge in multilingual pretrained language models, arXiv:2102.00894.
69. Sarkar, R., Mahinder, S., and KhudaBukhsh, A.R. (2020). The non-native speaker aspect: indian English in social media. In Proceedings of the Sixth Workshop on Noisy User-Generated Text, W-NUT@EMNLP 2020 Online, November 19, 2020 (Association for Computational Linguistics), pp. 61–70. https://doi.org/10.18653/v1/2020.wnut-1.9.
70. Nayak, P., and Mahanta, B. (2012). Women empowerment in India. Bull. Polit. Econ. *5*, 155–183.
71. Chen, M. (1995). A Matter of Survival: Women's Right to Employment in India and Bangladesh, *Vol. 38* (Clarendon Press).
72. Goyal, M., and Parkash, J. (2011). Women entrepreneurship in India-problems and prospects. Int. J. Multidisc. Res. *1*, 195–207.
73. Nigam, S. (2005). Understanding Justice Delivery System from the Perspective of Women Litigants as Victims of Domestic Violence in India (Specifically in the Context of Section 498-A, IPC) (Nigam Shalu), Occasional Paper (35).
74. Fiore, A.T., Taylor, L.S., Mendelsohn, G.A., and Hearst, M. (2008). Assessing attractiveness in online dating profiles. Proceedings of the SIGCHI Conference on Human Factors in Computing Systems, pp. 797–806.
75. Rose, J., Mackey-Kallis, S., Shyles, L., Barry, K., Biagini, D., Hart, C., and Jack, L. (2012). Face it: the impact of gender on social media images. Commun. Q. *60*, 588–607.
76. Otterbacher, J. (2015). Linguistic bias in collaboratively produced biographies: crowdsourcing social stereotypes? In Ninth International AAAI Conference on Web and Social Media.
77. Dlova, N.C., Hamed, S.H., Tsoka-Gwegweni, J., and Grobler, A. (2015). Skin lightening practices: an epidemiological study of South African women of African and Indian ancestries. Br. J. Dermatol. *173*, 2–9.
78. Chattopadhyay, S. (2019). Fair-Unfair: Prevalence of Colorism in Indian Matrimonial Ads and Married Women's Perceptions of Skin-Tone Bias in India, Ph.D. thesis (Bowling Green State University).
79. Madhukalya, A. (2020). These dialogues from bollywood blockbusters are so sexist that you'll want to pull your hair out. https://www.huffingtonpost.in/2016/09/26/these-dialogues-from-bollywood-blockbusters-are-so-sexist-that-y_a_21479215/.
80. Olcott, M. (1944). The caste system of India. Am. Sociol. Rev. *9*, 648–657.
81. LaFeber, W., and Abbott, B. (1972). America, Russia, and the Cold War, 1945-1975 (Wiley).
82. M. Lundberg, Bollywood images: the illusions and realities of arranged marriages, weddings, dowries, and attitudes toward the girl child in the lives of women in India, Augsburg Honors Rev. 6.
83. Diamond-Smith, N., Saikia, N., Bishai, D., and Canudas-Romo, V. (2020). What has contributed to improvements in the child sex ratio in select districts of India? a decomposition of the sex ratio at birth and child mortality. J. Biosoc. Sci. *52*, 27–36.
84. Firstpost (2014). Acid attack victim laxmi to receive international women of courage award - India News. https://www.firstpost.com/india/acid-attack-victim-laxmi-to-receive-international-women-of-courage-award-1418519.html.
85. Bender, E.M., Gebru, T., McMillan-Major, A., and Shmitchell, S. (2021). On the dangers of stochastic parrots: can language models be too big? In

CellPress
OPEN ACCESS

Proceedings of the 2021 ACM Conference on Fairness, Accountability, and Transparency, pp. 610–623.

86. P. Lison, J. Tiedemann, Opensubtitles2016: Extracting Large Parallel Corpora from Movie and TV Subtitles.Universitetsbiblioteket i Oslo

87. Greene, D. (2016). Rock, Counterculture and the Avant-Garde, 1966-1970: How the Beatles, Frank Zappa and the Velvet Underground Defined an Era (McFarland).

88. Athique, M., and Hill, D. (2007). Multiplex cinemas and urban redevelopment in India. Media Int. Aust. *124*, 108–118.

89. Pennington, J., Socher, R., and Manning, C. (2014). GloVe: global vectors for word representation. In Proceedings of the 2014 Conference on Empirical Methods in Natural Language Processing (EMNLP) (Association for Computational Linguistics), pp. 1532–1543. https://doi.org/10.3115/v1/D14-1162. https://aclanthology.org/D14-1162.

90. Mikolov, T., Sutskever, I., Chen, K., Corrado, G.S., and Dean, J. (2013). Distributed representations of words and phrases and their compositionality. In Advances in Neural Information Processing Systems, pp. 3111–3119.

Patterns, Volume *3*

Supplemental information

Gender bias, social bias, and representation: 70 years of B^{H}ollywood

Kunal Khadilkar, Ashiqur R. KhudaBukhsh, and Tom M. Mitchell

PRELIMINARY PREPRINT VERSION: DO NOT CITE
The AAAI Digital Library will contain the published
version some time after the conference.

An Unfair Affinity Toward Fairness: Characterizing 70 Years of Social Biases in B^{H}ollywood (Student Abstract)

Kunal Khadilkar[1] and Ashiqur R. KhudaBukhsh [1]

[1] Carnegie Mellon University
kkhadilk@cs.cmu.edu, akhudabu@cs.cmu.edu

Abstract

Bollywood, aka the Mumbai film industry, is one of the biggest movie industries in the world with a current movie market share of worth 2.1 billion dollars and a target audience base of 1.2 billion people. While the entertainment impact in terms of lives that Bollywood can potentially touch is mammoth, no NLP study on social biases in Bollywood content exists. We thus seek to understand social biases in a developing country through the lens of popular movies. Our argument is simple – popular movie content reflects social norms and beliefs in some form or shape. We present our preliminary findings on a longitudinal corpus of English subtitles of popular Bollywood movies focusing on (1) social bias toward a fair skin color (2) gender biases, and (3) gender representation. We contrast our findings with a similar corpus of Hollywood movies. Surprisingly, we observe that much of the biases we report in our preliminary experiments on the Bollywood corpus, also gets reflected in the Hollywood corpus.

Introduction

On a diachronic corpus of popular entertainment, what types of social biases can we analyze and detect? In this paper, we analyze a curated corpus of film subtitles from the Bollywood and Hollywood film industry, spread over 70 years and present our preliminary findings on three aspects: (1) social bias toward a fair skin color; (2) gender biases; and (3) gender representation. While studies analyzing gender stereotypes across different languages (Lewis and Lupyan 2020) and detecting bias in word embeddings (Garg et al. 2018) exist, Bollywood in NLP literature has received little focus (Khan and Taylor 2018; Madaan et al. 2018) and no existing work has considered the rich data set of subtitles contrasting the findings with a Hollywood subtitles corpus.

In Figure 1, what do the prominent presence of negative adjectives such as `wanton` in old Bollywood movies (see, Figure 1(a)) juxtaposed with the presence of positive verbs such as `respect` (see, Figure 1(b)) tell us? In this paper, we explore a recent technique (Palakodety, KhudaBukhsh, and Carbonell 2020) used to mine political insights in a novel context of uncovering social biases. Through a series of cloze tests (Taylor 1953) on a language model (Devlin et al. 2019) fine-tuned on our data sets, we present our preliminary findings.

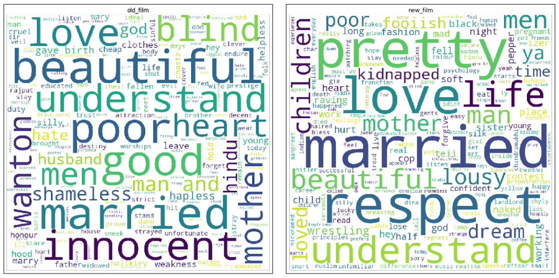

(a) Old Bollywood (b) Recent Bollywood

Figure 1: Word cloud visualizations for the five word contexts containing utterances of 'woman/women'.

Our data sets: We construct the following two data sets.
1. Bollywood movies, $\mathcal{D}_{bolly}$: We consider movies spanning seven decades (1950–2020). For each decade, we retrieved subtitles (Lison and Tiedemann 2016) of 40 top-grossing movies (280 total films), based on earnings documented by IMDb (Internet Movie Database).
2. Hollywood movies, $\mathcal{D}_{holly}$: Similarly, we considered 40 top-grossing movies from each of the seven decades (280 total films). Overall, $\mathcal{D}_{bolly}$ and $\mathcal{D}_{holly}$ consist of 542,768 and 488,498 dialogues, respectively.

For a subset of our analyses, we divide our corpus into two buckets: (1) Films from 1950 to 1970 ($\mathcal{D}^{old}_{bolly}$ and $\mathcal{D}^{old}_{holly}$); and (2) Films from 2000 to 2020 ($\mathcal{D}^{new}_{bolly}$ and $\mathcal{D}^{new}_{holly}$).

Results

Pronouns as a Proxy for Representation: Following extensive literature on gendered pronouns' relative distributions and their implications (Twenge, Campbell, and Gentile 2012), we consider a simple measure of gender representation: relative occurrence of pronouns of each gender (Men: he, him. Women: she, her). Let $\mathcal{N}_w$ denote the number of times a token w appears in a corpus. We define *Male Pronoun Ratio (MPR)* as follows:
$MPR = \frac{\mathcal{N}_{he}+\mathcal{N}_{him}}{\mathcal{N}_{he}+\mathcal{N}_{him}+\mathcal{N}_{she}+\mathcal{N}_{her}} * 100$. Figure 2 plots *MPR* of our decade-wise movie data sets and contrasts with *MPR* computed using Google Ngrams. Our results indicate that even now, both Bollywood and Hollywood exhibit compa-

Copyright © 2021, Association for the Advancement of Artificial Intelligence (www.aaai.org). All rights reserved.

Probe	$\texttt{BERT}_{\textbf{base}}$	$\texttt{BERT}_{\mathcal{D}^{old}_{bolly}}$	$\texttt{BERT}_{\mathcal{D}^{new}_{bolly}}$	$\texttt{BERT}_{\mathcal{D}^{old}_{holly}}$	$\texttt{BERT}_{\mathcal{D}^{new}_{holly}}$
$cloze_1$	soft, beautiful, pale, tanned, smooth	fair, no, pale, tanned, tan	fair, tanned, golden, smooth, pale	fair, pale, blue, golden, gold	fair, pale, tanned, golden, dark
$cloze_2$	woman, man, widow, *doctor*, slave, *soldier*, bachelor, *merchant*, *farmer*, *lawyer*, servant	prostitute, servant, woman, slave, bachelor, *doctor*, *lawyer*, man, widow, maid, *worker*	*doctor*, woman, servant, *lawyer*, maid, man, *nurse*, *teacher*, *gardener*, lady, hindu	woman, slave, servant, *nurse*, lady, man, *teacher*, *lawyer*, peasant, maid, wife	woman, *lawyer*, *doctor*, *nurse*, *teacher*, man, *writer*, *secretary*, prostitute, professional, *carpenter*
$cloze_3$	man, *soldier*, gentleman, *farmer*, *merchant*, woman, slave, bachelor, *doctor*, *carpenter*, servant	man, *gentleman*, bachelor, *lawyer*, servant, *doctor*, *farmer*, *worker*, *craftsman*, slave, criminal	*doctor*, *lawyer*, *policeman*, man, *farmer*, bachelor, *gardener*, servant, *soldier*, *mechanic*, *builder*	*carpenter*, *policeman*, *lawyer*, *soldier*, *farmer*, *gentleman*, servant, man, peasant, slave, *doctor*	man, *lawyer*, *soldier*, *doctor*, *carpenter*, *gentleman*, *farmer*, *clergyman*, *writer*, *craftsman*, *minister*

Table 1: Cloze test results. Predicted tokens are ranked by decreasing probability. Positive and negative words are italicized and underlined, respectively. Our completion results using `BERT` indicate presence of social and gender biases.

rable skew in gendered pronoun usage.

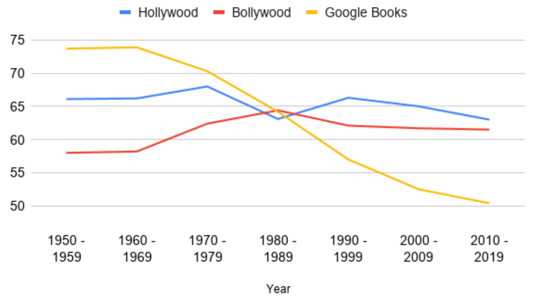

Figure 2: MPR in $\mathcal{D}_{bolly}$ and $\mathcal{D}_{holly}$

Cloze Test Using Language Models: When presented with a sentence (or a sentence stem) with a missing word, a cloze task (Taylor 1953) is essentially a fill-in-the-blank task. For instance, in the following cloze task: *In the* `[MASK]`, *it is very sunny*, `summer` is a likely completion for the missing word. Given a cloze test, `BERT`, a well-known language model (Devlin et al. 2019), outputs a series of token ranked by probability. In fact, in the above cloze test, the top three tokens (ranked by probability) predicted by $\texttt{BERT}_{base}$ are: summer, winter and spring. Recent lines of research has explored `BERT`'s masked query prediction for (1) knowledge base extraction (Petroni et al. 2019) and (2) mining political insights (Palakodety, KhudaBukhsh, and Carbonell 2020).

Following (Palakodety, KhudaBukhsh, and Carbonell 2020), we fine-tune `BERT` on four sub-corpora: $\mathcal{D}^{old}_{bolly}$, $\mathcal{D}^{old}_{holly}$,$\mathcal{D}^{old}_{bolly}$, and $\mathcal{D}^{old}_{holly}$. We denote the pretrained `BERT` model as $\texttt{BERT}_{base}$ and a fine-tuned `BERT` on corpus $\mathcal{D}$ as $\texttt{BERT}_{\mathcal{D}}$. Out of a thorough analysis with several cloze tests with phrase variations, due to paucity of space, in Table 1 we report only three such results for the following cloze tests: (1) *A beautiful woman should have* `[MASK]` *skin*. (*cloze1*); (2) *A woman should be a* `[MASK]` *by occupation*. (*cloze2*); and (3) *A man should be a* `[MASK]` *by occupation* (*cloze3*).

Summary of findings: Given cultural and social bias toward fair skin prevalent in India for years (Karan 2008), it is not surprising that Bollywood exhibits an affinity toward fairness. However, our analysis indicates Hollywood also shows bias toward lighter skin color. Further, we observe strong presence of gender bias with masked sentences involving men predominantly receiving positive completions in comparison with women (see, Table 1). Future directions include (1) tracking moral sentiment; (2) exploring debiasing techniques; and (3) analyzing representation of other minorities.

References

Devlin, J.; Chang, M.-W.; Lee, K.; and Toutanova, K. 2019. BERT: Pre-training of Deep Bidirectional Transformers for Language Understanding. In *NAACL-HLT*, 4171–4186.

Garg, N.; Schiebinger, L.; Jurafsky, D.; and Zou, J. 2018. Word embeddings quantify 100 years of gender and ethnic stereotypes. *Proceedings of the National Academy of Sciences* 115(16): E3635–E3644.

Karan, K. 2008. Obsessions with fair skin: Color discourses in Indian advertising. *Advertising & society review* 9(2).

Khan, S.; and Taylor, L. 2018. Gender Policing in Mainstream Hindi Cinema: A Decade of Central Female Characters in Top-Grossing Bollywood Movies. *International Journal of Communication* 12: 22.

Lewis, M.; and Lupyan, G. 2020. Gender stereotypes are reflected in the distributional structure of 25 languages. *Nature human behaviour* 1–8.

Lison, P.; and Tiedemann, J. 2016. Opensubtitles2016: Extracting large parallel corpora from movie and tv subtitles .

Madaan, N.; Mehta, S.; Agrawaal, T.; Malhotra, V.; Aggarwal, A.; Gupta, Y.; and Saxena, M. 2018. Analyze, detect and remove gender stereotyping from bollywood movies. In *Conference on Fairness, Accountability and Transparency*, 92–105.

Palakodety, S.; KhudaBukhsh, A. R.; and Carbonell, J. G. 2020. Mining Insights from Large-Scale Corpora Using Fine-Tuned Language Models. In *ECAI 2020*, 1890–1897.

Petroni, F.; Rocktäschel, T.; Riedel, S.; Lewis, P.; Bakhtin, A.; Wu, Y.; and Miller, A. 2019. Language Models as Knowledge Bases? In *EMNLP-IJCNLP*, 2463–2473.

Taylor, W. L. 1953. "Cloze procedure": A new tool for measuring readability. *Journalism quarterly* 30(4): 415–433.

Twenge, J. M.; Campbell, W. K.; and Gentile, B. 2012. Male and female pronoun use in US books reflects women's status, 1900–2008. *Sex roles* 67(9-10): 488–493.
